# Deep immunophenotyping reveals circulating activated lymphocytes in individuals at risk for rheumatoid arthritis

**DOI:** 10.1172/JCI185217

**Published:** 2025-03-17

**Authors:** Jun Inamo, Joshua Keegan, Alec Griffith, Tusharkanti Ghosh, Alice Horisberger, Kaitlyn Howard, John F. Pulford, Ekaterina Murzin, Brandon Hancock, Salina T. Dominguez, Miranda G. Gurra, Siddarth Gurajala, Anna Helena Jonsson, Jennifer A. Seifert, Marie L. Feser, Jill M. Norris, Ye Cao, William Apruzzese, S. Louis Bridges, Vivian P. Bykerk, Susan Goodman, Laura T. Donlin, Gary S. Firestein, Joan M. Bathon, Laura B. Hughes, Andrew Filer, Costantino Pitzalis, Jennifer H. Anolik, Larry Moreland, Nir Hacohen, Joel M. Guthridge, Judith A. James, Carla M. Cuda, Harris Perlman, Michael B. Brenner, Soumya Raychaudhuri, Jeffrey A. Sparks, The Accelerating Medicines Partnership RA/SLE Network, V. Michael Holers, Kevin D. Deane, James Lederer, Deepak A. Rao, Fan Zhang

**Affiliations:** 1 Division of Rheumatology and; 2 Department of Biomedical Informatics, Center for Health Artificial Intelligence, University of Colorado School of Medicine, Aurora, Colorado, USA.; 3 Department of Surgery, Brigham and Women’s Hospital and Harvard Medical School, Boston, Massachusetts, USA.; 4 Department of Biostatistics & Informatics, University of Colorado School of Medicine, Aurora, Colorado, USA.; 5 Department of Medicine, Division of Rheumatology, Inflammation, and Immunity, Brigham and Women’s Hospital and Harvard Medical School, Boston, Massachusetts, USA.; 6 Lausanne University Hospital, University of Lausanne, Lausanne, Switzerland.; 7 Department of Medicine, Division of Rheumatology and; 8 Department of Preventive Medicine, Division of Biostatistics and Informatics, Northwestern University, Chicago, Illinois, USA.; 9 Broad Institute of MIT and Harvard, Cambridge, Massachusetts, USA.; 10 Department of Epidemiology, Colorado School of Public Health, Aurora, Colorado, USA.; 11 The list of the Accelerating Medicines Partnership: Rheumatoid Arthritis and Systemic Lupus Erythematosus (AMP RA/SLE) Program members is provided in Supplemental Acknowledgments.; 12 Department of Medicine, Hospital for Special Surgery, New York, New York, USA.; 13 Weill Cornell Medical College, New York, New York, USA.; 14 Division of Rheumatology, Allergy, and Immunology, UCSD, La Jolla, California, USA.; 15 Department of Medicine, Division of Rheumatology, Columbia University, New York, New York, USA.; 16 Department of Medicine, Division of Clinical Immunology and Rheumatology, University of Alabama at Birmingham Medicine, Birmingham, Alabama, USA.; 17 Rheumatology Research Group, Institute for Inflammation and Ageing, University of Birmingham, Birmingham, United Kingdom.; 18 National Institute for Health and Care Research (NIHR) Birmingham Biomedical Research Centre and Clinical Research Facility, University of Birmingham and University Hospitals Birmingham Foundation Trust, Birmingham, United Kingdom.; 19 Centre for Experimental Medicine and Rheumatology, William Harvey Research Institute, Queen Mary University of London and Barts NIHR BRC & NHS Trust, London, United Kingdom.; 20 Department of Biomedical Sciences, Humanitas University, and Humanitas Research Hospital, Milan, Italy.; 21 Division of Allergy, Immunology and Rheumatology, University of Rochester Medical Center, Rochester, New York, USA.; 22 Arthritis & Clinical Immunology Research Program, Oklahoma Medical Research Foundation, Oklahoma City, Oklahoma, USA.; 23 Center for Data Sciences,; 24 Department of Medicine, Division of Genetics, and; 25 Department of Biomedical Informatics, Brigham and Women’s Hospital and Harvard Medical School, Boston, Massachusetts, USA.

**Keywords:** Autoimmunity, Immunology, Arthritis, Bioinformatics, Rheumatology

## Abstract

Rheumatoid arthritis (RA) is a systemic autoimmune disease currently with no universally highly effective prevention strategies. Identifying pathogenic immune phenotypes in at-risk populations prior to clinical onset is crucial to establishing effective prevention strategies. Here, we applied multimodal single-cell technologies (mass cytometry and CITE-Seq) to characterize the immunophenotypes in blood from at-risk individuals (ARIs) identified through the presence of serum antibodies against citrullinated protein antigens (ACPAs) and/or first-degree relative (FDR) status, as compared with patients with established RA and people in a healthy control group. We identified significant cell expansions in ARIs compared with controls, including CCR2^+^CD4^+^ T cells, T peripheral helper (Tph) cells, type 1 T helper cells, and CXCR5^+^CD8^+^ T cells. We also found that CD15^+^ classical monocytes were specifically expanded in ACPA-negative FDRs, and an activated PAX5^lo^ naive B cell population was expanded in ACPA-positive FDRs. Further, we uncovered the molecular phenotype of the CCR2^+^CD4^+^ T cells, expressing high levels of Th17- and Th22-related signature transcripts including *CCR6*, *IL23R*, *KLRB1*, *CD96*, and *IL22*. Our integrated study provides a promising approach to identify targets to improve prevention strategy development for RA.

## Abstract 2

This study identifies specific immune cell expansions in individuals at risk of rheumatoid arthritis, offering potential targets for effective prevention strategies.

## Graphical abstract

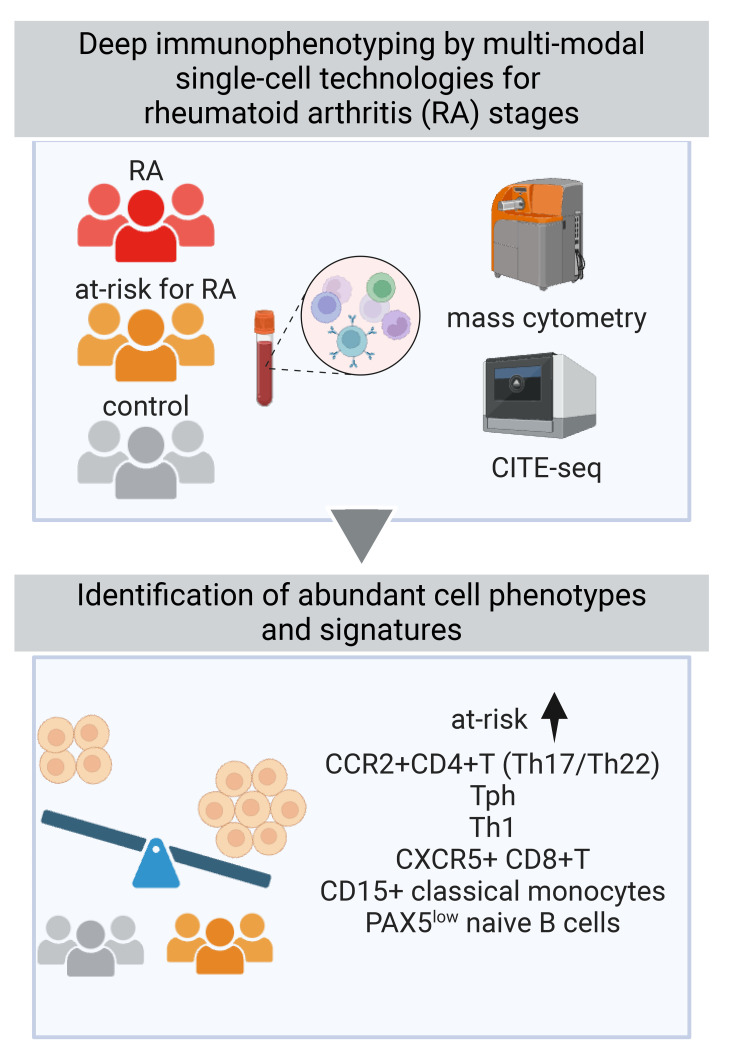

## Introduction

Rheumatoid arthritis (RA) is an autoimmune disease that affects 0.8%–1.0% of the population (1, 2). Although the presence of inflammatory arthritis is the hallmark of clinical RA, diagnosis and treatments are usually delayed, and no cure has been found (3). Recent studies have shown that in seropositive RA there is a prolonged pre-RA phase characterized by blood elevations of biomarkers (4–6), including antibodies against citrullinated protein antigens (ACPAs), prior to the onset of “clinical RA” (i.e., the first appearance of inflammatory arthritis) (7, 8).

Notably, individuals can be defined for studies as being at high risk for future RA due to being a first-degree relative (FDR) of a patient with RA and/or having elevations of ACPAs in the peripheral blood (9–12); notably, these individuals may be termed “at-risk individuals” (ARIs). In particular, serum elevations of ACPAs are highly predictive of the future development of RA (13–16), and multiple studies of ACPA^+^ ARIs have been performed to evaluate possible preventive strategies. While trials of corticosteroids, atorvastatin, methotrexate, and B cell–depleted therapy have not conclusively prevented progression to clinical RA, rituximab delayed the onset of inflammatory arthritis, and methotrexate was associated with improved symptoms and measure of disease activity even if RA developed; furthermore, in 2 studies, abatacept reduced rates of progression to clinical RA within the trial periods and beyond. In aggregate, these findings suggest that there is a potential window of opportunity for preventing or modulating the course of the disease (17–23).

The mechanisms that drive autoimmunity in at-risk states are still unclear, but likely involve a complex interplay between genetics, environmental factors, mucosal endotypes, and immunophenotypes (24–29). Thus, investigating the spectrum of molecular and cellular changes in individuals who are in the at-risk state is key to identifying predictive markers and phenotypes to further develop accurate prediction models for future RA, and identify targets for preventive interventions (1, 24, 29, 30). There are an increasing number of studies focused on characterizing immunophenotypes and biomarkers in ARIs (31–36). Several immune phenotyping studies in blood or lymphoid tissues from ARIs have revealed alterations, including naive CD4^+^ T cells (37, 38), CD8^+^ T cells (39), natural killer (NK) cell subsets (40), T follicular helper (Tfh) cells (41), and activated CD4^+^CD69^+^ (42) and CD8^+^CD69^+^ T cells (43). Our recent work has identified an expansion of T cells reactive to citrullinated cartilage intermediate layer protein 1 with Th1, Th17, and T stem cell memory-like phenotypes (44). In addition, our group consistently observed expansion of HLA-DR^+^ T peripheral helper (Tph) cells and CXCR5^–^CD11c^–^CD38^+^ naive B cells in ACPA^+^ ARIs in 2 independent cohorts (45). However, a comprehensive, unbiased assessment of the peripheral immune landscape in ARIs using highly multiplexed techniques has been lacking. We reasoned that applying high-dimensional cytometric immunophenotyping combined with computational integration strategies in a systematic manner would reveal additional relevant features in RA immune activation that would inform the understanding of disease pathogenesis.

Multi-institutional studies using single-cell technologies are advancing our understanding of autoimmune disease heterogeneity, in part through the increased power of uniform analyses of pooled cohorts (46–49). Our recent work of harmonizing single-cell transcriptomics, mass cytometry, and single-cell multimodal cellular indexing of transcriptomes and epitopes by sequencing (CITE-Seq) from multiple clinical sites has uncovered key immune cell populations in inflamed synovium from patients with established RA (46, 50). We and others have developed robust single-cell integration methods and identified specific RA-relevant immune populations (47, 51–55), including our recently identified GZMK^+^ CD8^+^ T cells (56), previously identified Tph and Tfh cells (46, 57), proinflammatory myeloid cells like IL-1B^+^HBEGF^+^ (46, 58), IFN- and TNF-driven CXCL10^+^CCL2^+^ macrophages (47), and NR4A^+^ B cells (59). However, whether any of these pathogenic populations in the inflamed tissues are already present and altered in the circulation during the pre-RA phase of disease is unclear (47, 51–55). Such populations may represent key treatment targets to prevent development of clinical RA.

Under the Studies of the Etiologies of Rheumatoid Arthritis (SERA) umbrella, as well as through the Accelerating Medicines Partnership Rheumatoid Arthritis and Systemic Lupus Erythematosus (AMP RA/SLE) Network, multi-institutional cohorts of ARIs, patients with established RA, and relevant controls have been established to study the natural history of RA (31–36). Here, we performed deep single-cell immunophenotyping using large mass cytometry data to characterize peripheral blood mononuclear cell (PBMC) alterations in ARIs compared with patients with established RA and healthy controls, and validated the results using CITE-Seq. Using our integrative and classification strategies, we identified multiple immune cell populations expanded in blood of various ARIs based on the presence of ACPAs and/or FDR status. Our computational strategies and immunophenotypic findings define specific features of immune dysregulation in preclinical RA and nominate new potential targets for immunophenotype-based preventive strategies.

## Results

### Single-cell proteomic profiling of mononuclear cells defines differential immune cell abundance in ARIs.

**Figure 1:**
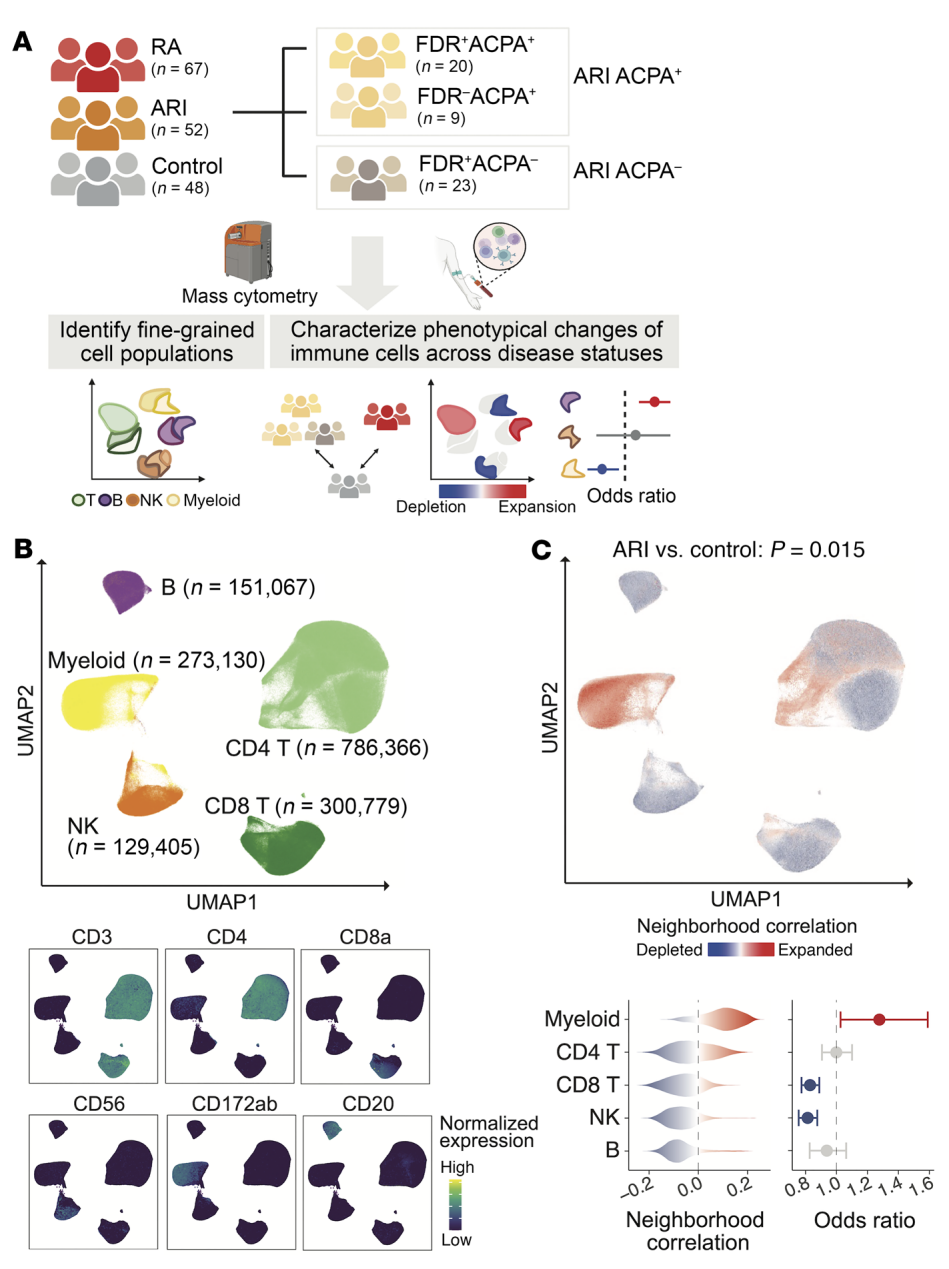
Overview of mass cytometry pipeline and cell type abundance analysis for at-risk individuals using all mononuclear cells. (**A**) Description of study design regarding patient recruitment, clinical classification, and computational strategies. (**B**) Identified major immune cell types among all mononuclear cells and canonical protein expression in uniform manifold approximation and projection (UMAP). (**C**) At-risk individual (ARI) associations compared with controls for all mononuclear cells. *P* value was generated from covarying neighborhood analysis (CNA). Cells in UMAP are colored for expansion (red) or depletion (blue) in ARIs. For each cell type, distributions of ARI-associated cell neighborhood correlations and odds ratios with 95% confidence intervals are shown. All the ARI association testing was adjusted for age and sex.

We analyzed PBMCs from ARIs and established RA patients who were enrolled in the AMP RA/SLE Network using mass cytometry. We then applied computational algorithms to identify covarying phenotypical changes for cellular and molecular heterogeneity across different ARIs and clinical groups (Figure 1A and Supplemental Table 1; supplemental material available online with this article; https://doi.org/10.1172/JCI185217DS1). Sensitivity analyses confirmed the stability of immune cell clusters across various downsampling parameters (Supplemental Figure 1). We first investigated all mononuclear cells from 167 individuals to characterize the landscape of immune cell heterogeneity, and then quantified the altered cell abundance across differential clinical phenotypes. We defined 4 major immune cell types, T cells, myeloid cells, B cells, and NK cells, based on canonical protein markers and projected them into low-dimensional space (Figure 1B). To identify significant shifts of major cell type abundance between ARIs and controls, we applied complementary computational strategies, including cluster-based mixed-effects modeling of associations of single cells (MASC) (53) accounting for covariates age and sex, and cluster-free covarying neighborhood analysis (CNA) (54) that identifies dominant covarying cell type abundance while accounting for covariates age and sex. We observed significantly expanded myeloid cells in ARIs (*P* = 0.015, odds ratio [OR] = 1.28) (Figure 1C). Conversely, CD8^+^ T cells (OR = 0.83) and NK cells (OR = 0.81) were depleted in ARIs compared with controls. We also observed heterogeneity within CD4^+^ T cells and their variable association levels, which suggested a need for fine-grained cell type–specific analysis.

### Cell type–specific analysis reveals fine-grained cell subpopulations.

**Figure 2:**
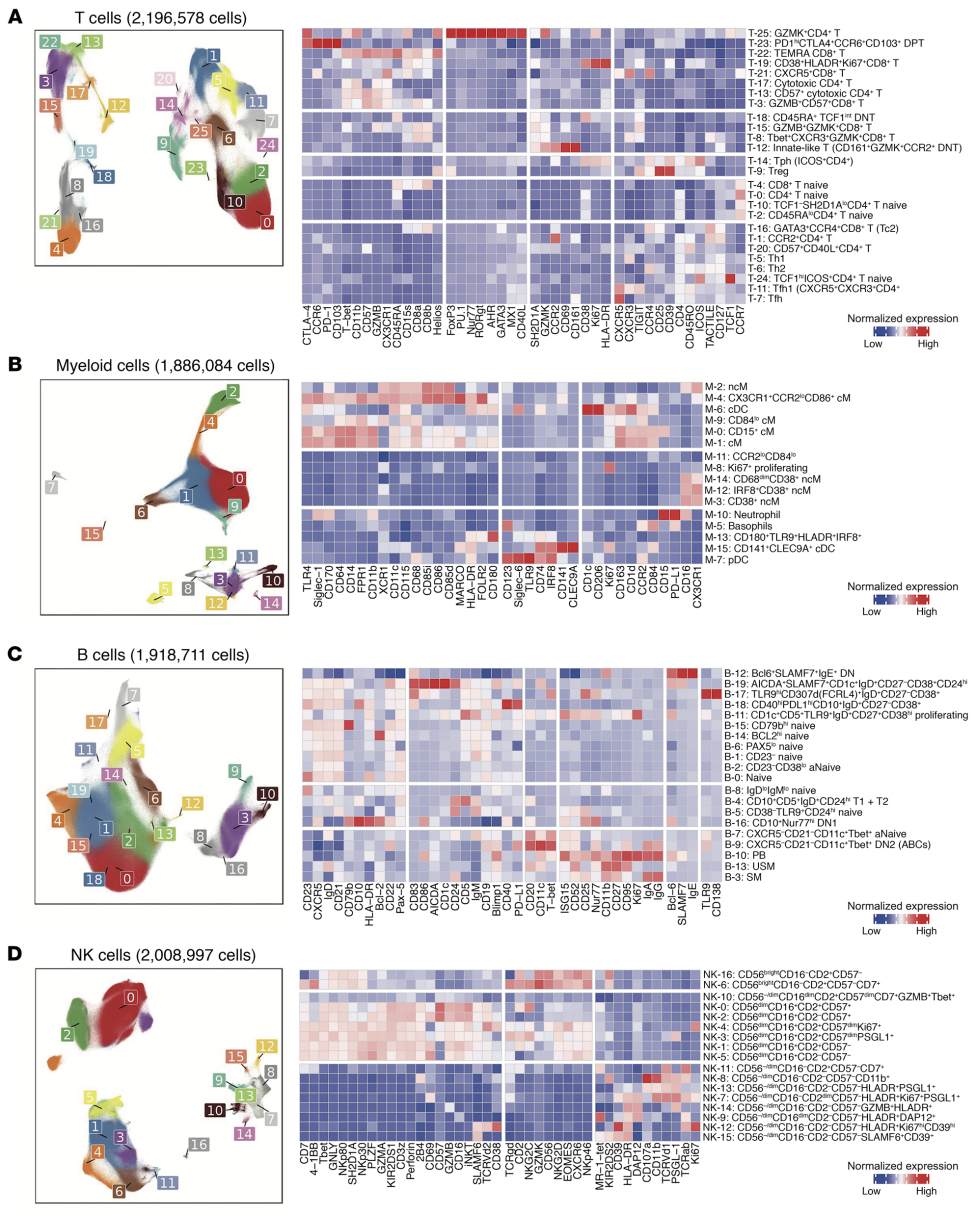
Cell type–specific clustering analysis reveals 79 distinct cell states. (**A**–**D**) Cell type–specific immune proteomic reference colored by fine-grained cell states in the UMAP space. For each cell type, the heatmap shows the average expression distributions of key variable proteins in each cluster across samples, scaled within each cell cluster. Clusters are ordered by protein expression pattern using hierarchical clustering.

For each major immune cell type, we defined fine-grained cell states based on expression of 48 protein markers and quantified cluster abundances and phenotypical changes (Figure 2, Supplemental Figure 2, and Supplemental Table 2). We identified 79 immune cell clusters in total, including 26 T cell clusters, 16 myeloid clusters, 20 B cell clusters, and 17 NK cell clusters. We described differentially expressed proteins for each cluster and summarized the statistics (Supplemental Table 3). The T cells clustered broadly into CD4^+^ and CD8^+^ T cell subsets (Figure 2A and Supplemental Figure 3). Among the CD4^+^ T cells, naive T cells (T-0, T-2) separated from memory cell populations, and a distinct population of regulatory T cells (T-9) marked by FoxP3 and CD25 clustered separately. The memory CD4^+^ T cells included CXCR5^+^ Tfh cells (T-7, T-11) as well as a cluster of PD-1^+^ICOS^+^ Tph cells (T-14), a B cell helper population highly enriched in RA joints (46, 57). CD8^+^ T cells also separated in naive and effector-memory subsets, with the memory cells segregating into distinct granzyme B^+^ and granzyme K^+^ subpopulations (T-3, T-8, T-15), as was also observed among CD8^+^ T cells in RA synovium (56, 60).

Among myeloid cells, we observed classical monocytes (M-0, M-1, M-4, M-9) and nonclassical monocytes (M-2), as well as conventional dendritic cell populations (M-6, M-15), and a separate plasmacytoid dendritic cell cluster (M-7) (Figure 2B and Supplemental Figure 4). In addition, we observed a small population of neutrophils (M-10), likely representing low-density granulocytes, as well as a small population of basophils (M-5). B cells predominantly segregated into IgD^+^ naive (B-0-2, B-5-8, B-14, and B-15) and CD27^+^ memory B populations (B-3, B-13), while distinct populations of CXCR5^lo^CD21^lo^CD11c^+^ activated naive B cells (B-7) and CD11c^+^ age-associated B cells (DN2) (B-9) clustered separately (Figure 2C and Supplemental Figure 5). NK cells separated predominantly into CD56^dim^ and CD56^bright^ populations (Figure 2D and Supplemental Figure 6). In all, many of the disease-relevant immune cell phenotypes were detectable in the peripheral blood from the ARIs.

### Unique immunophenotypes characterize ARI subpopulations.

**Figure 3:**
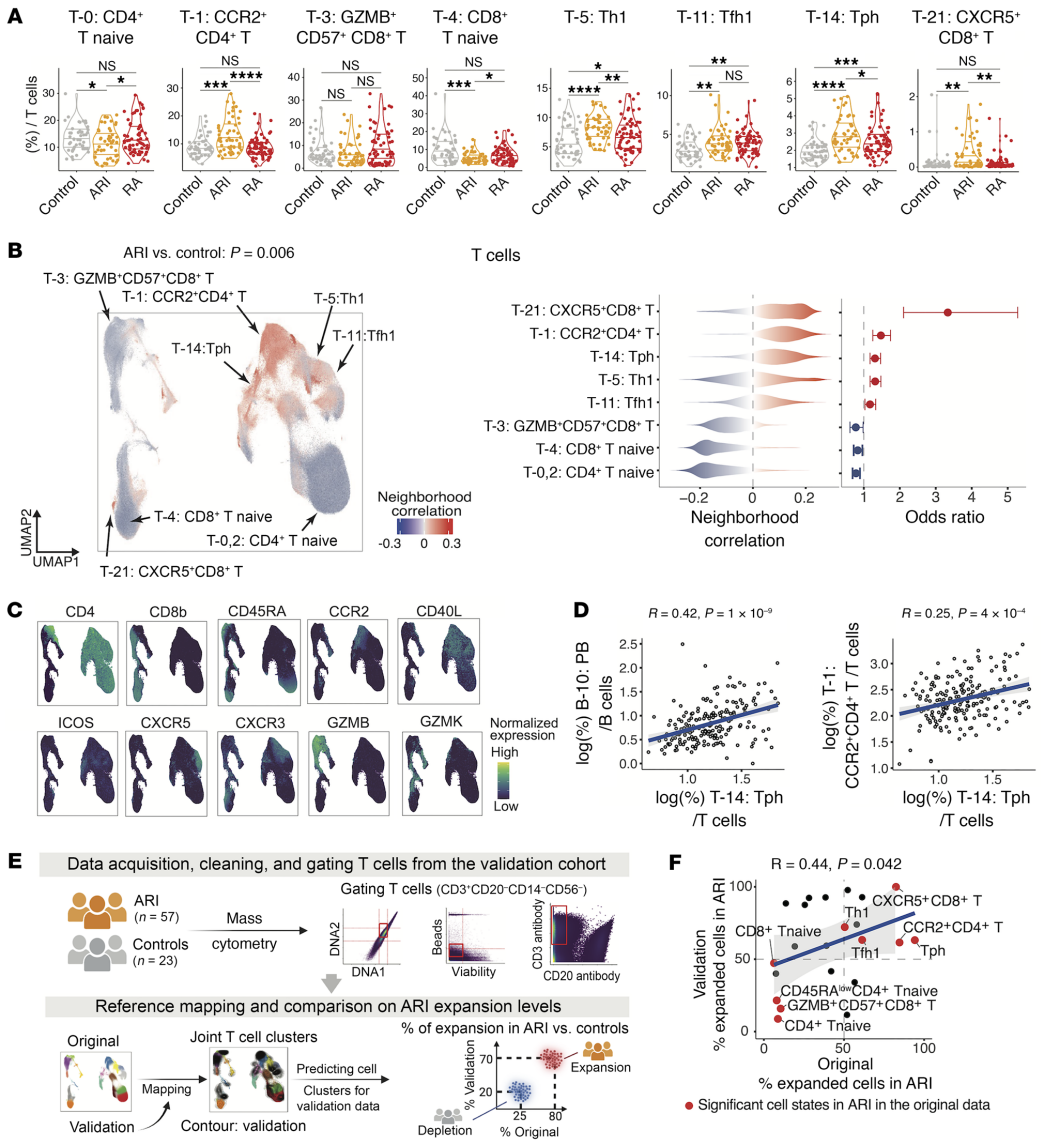
Identification of specific T cell populations that were associated with ARI. (**A**) Distribution of frequencies of cell types identified as ARI-related cell types in **B**. *P < 0.05, **P < 0.01, ***P < 0.001, ****P < 0.0001 by 2-sided Wilcoxon’s test. (**B**) Left: Associations of T cell neighborhoods with ARIs versus controls. For all CNA-based association results, cells in UMAP are colored in red (expansion) or blue (depletion), and *P* value is shown as well. Distributions of cell neighborhood correlations (middle) and odds ratios (right) are shown. Error bars for odds ratios represent 95% confidence intervals. (**C**) Expression of selected surface proteins within T cells is colored from dark blue (low) to green (high). (**D**) Scatterplot of cell type abundance correlations across individuals. (**E**) Description of the validation dataset and analytical strategies, including reference mapping to the original T cell clusters, association test using CNA, and comparison of the proportion of expanded cells (neighborhood correlation > 0) in ARIs (vs. control) between 2 independent datasets by clusters. (**F**) Scatterplot of the proportion of expanded cells in ARIs by clusters, with *x* axis for the original T cell panel and *y* axis for the validation dataset. Red dots represent significant cell clusters in the original T cell panel. All statistical association tests were adjusted for age and sex. Correlation coefficients and *P* values were obtained from Spearman’s correlation test.

To identify disease-relevant immune cell phenotypes, we investigated the abundance shift of different immune cell subsets according to clinical groups. This analysis revealed the most expanded subsets in ARIs within the T cell compartment, including CCR2^+^CD4^+^ T cells (T-1), Th1 cells (T-5), Tfh1 cells (T-11), Tph cells (T-14), and a distinct subset of CXCR5^+^CD8^+^ T cells (T-21) (Figure 3A). To systematically quantify cell abundances and their differences between ARIs and controls, we identified expanded cell neighborhoods within ARIs (*n* = 52) compared with healthy controls (*n* = 48) accounting for technical batches and demographic variables, including clinical site, age, and sex (Methods). This integrative approach not only gains power but also further evaluates the specific at-risk status findings in a more generalized manner. We used this approach to identify specific immune phenotypes that are altered in ARIs compared with controls in T, myeloid, B, and NK cells, respectively, as follows.

Within T cells, we observed skewed cell type abundances between ARIs and controls (*P* = 0.006) (Figure 3, B and C, and Supplemental Tables 4 and 5). Specifically, cell neighborhoods among CCR2^+^CD4^+^ T cells (T-1), Th1 (T-5), Tfh1 (T-11), Tph (T-14), and CXCR5^+^CD8^+^ T (T-21) were expanded in ARIs, while both CD4^+^ and CD8^+^ naive T cells (T-0, T-2, and T-4) were depleted. The CXCR5^+^CD8^+^ T (T-21) cluster was the most expanded cluster in ARIs (OR = 3.33). This T cell population expressed high levels of chemokine receptors, but the cell frequency was relatively small (2,506 cells in blood of tested ARIs and controls) with wide confidence intervals (2.11 to 5.28). Interestingly, the second most expanded T cell population was the CCR2^+^CD4^+^ T (T-1) cluster (OR = 1.47), a relatively large cluster (113,775 cells). In contrast, the GZMB^+^ effector CD8^+^ T cell cluster (T-3) was depleted, as were 3 naive clusters (T0, T-2, T-4). Further, we investigated correlations between cell cluster abundances (Supplemental Figure 7) and found that the abundance of plasmablasts (PBs; B-10) and that of Tph cells were significantly correlated (*R* = 0.42, *P* = 1 × 10^–9^) (Figure 3D), which further supports the hypothesis that Tph cells promote PBs in inflammatory diseases (61, 62). Intriguingly, the cell abundances of CCR2^+^CD4^+^ T and Tph cells were significantly correlated (*R* = 0.25, *P* = 4 × 10^–4^) (Figure 3D). To validate our findings, we analyzed an independent mass cytometry dataset for T cells, which we generated from an independent cohort with ARIs (*n* = 57) and controls (*n* = 23) as a validation cohort. To identify common cell state clusters, we mapped cells from our validation cohort to our original T cell reference for comparative analysis, and then quantified the expansion and depletion of these T cell clusters in the validation dataset (Figure 3E, Supplemental Figure 8, Supplemental Tables 6 and 7, and Methods). Notably, we observed concordant expansion of several T cell clusters in ARIs between 2 independent cohorts, including CCR2^+^CD4^+^ T, Tph, Th1, Tfh1, and CXCR5^+^CD8^+^ T cells (Figure 3F). Moreover, we reanalyzed a single-cell multimodal dataset from RA synovial biopsies (50). We found that *CCR2* was expressed on multiple T cell populations infiltrating the RA synovium, including Tph cells, CD4^+^CD161^+^ memory T cells, and CD4^+^IL-17R^+^CCR5^+^ T cells (63) (Supplemental Figure 9).

**Figure 4:**
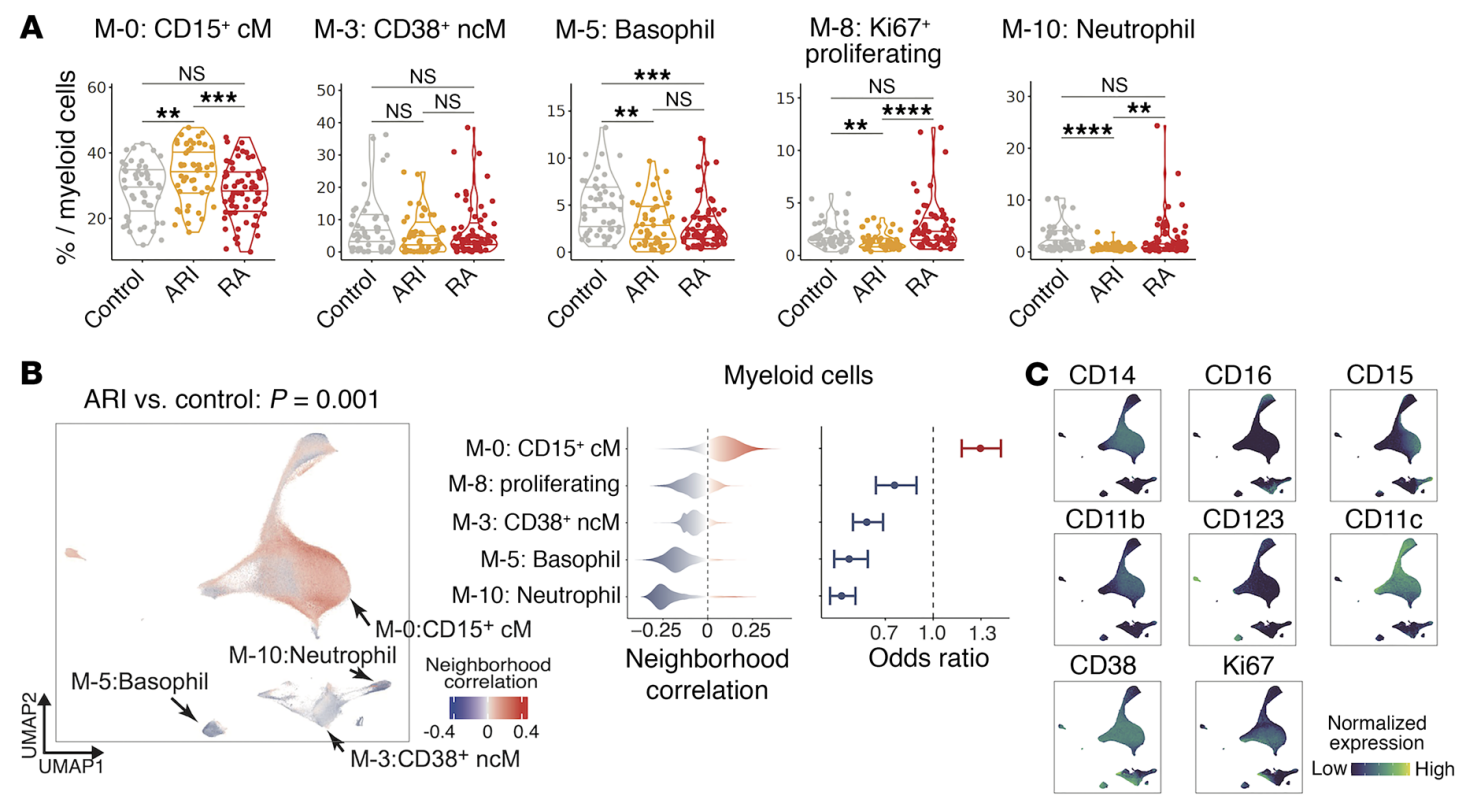
Identification of different myeloid cell populations that were associated with ARIs. (**A**) Distribution of frequencies of cell types identified as ARI-related cell types in **B**. **P < 0.01, ***P < 0.001, ****P < 0.0001 by 2-sided Wilcoxon’s test. (**B**) Associations of myeloid cell neighborhoods with ARIs versus controls (left), distributions of cell neighborhood correlations (middle), and odds ratios (right) are shown. (**C**) Expression of selected surface proteins within myeloid cells is colored from dark blue (low) to green (high).

**Figure 5:**
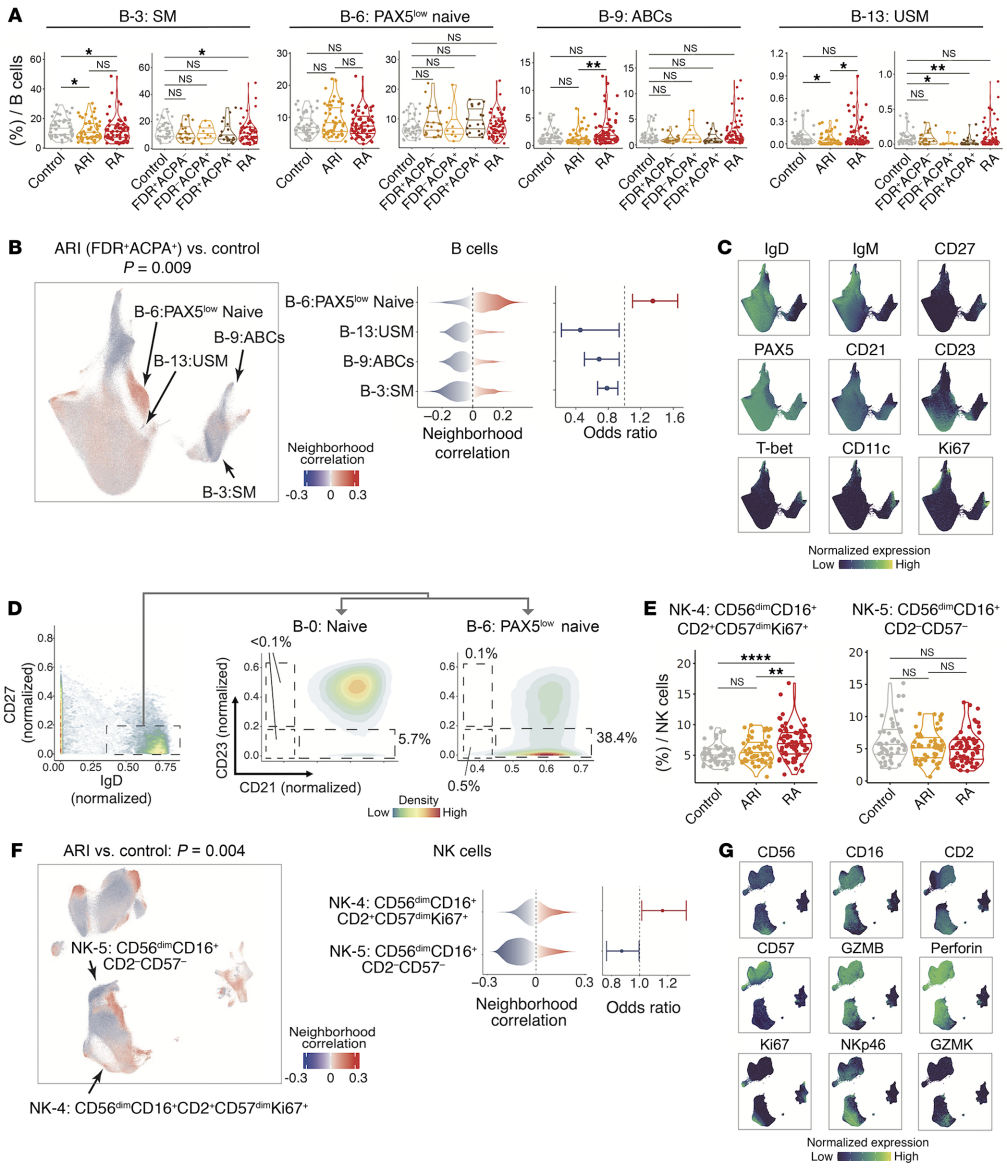
Identification of different B cell and NK cell populations that were associated with ARIs. (**A**) Distribution of frequencies of cell types for B cell subsets that were identified as ARI-related cell types in **B**. *P < 0.05, **P < 0.01 by 2-sided Wilcoxon’s test. (**B**) Associations of B cell neighborhoods with ARIs versus controls (left), distributions of cell neighborhood correlations (middle), and odds ratios (right) are shown. (**C**) Expression of selected surface proteins within B cells. (**D**) Distributions of activation marker (CD21 and CD23) antibody staining in the conventional naive B cell cluster (B-0) and the PAX5^lo^ naive B cell cluster (B-6). Low expression of CD21 and/or CD23 indicates activated B cells. (**E**) Distribution of frequencies of cell types for NK cell subsets that were identified as ARI-related cell types in **F**. **P < 0.01, ****P < 0.0001 by 2-sided Wilcoxon’s test. (**F**) Associations of NK cell neighborhoods with ARIs versus controls (left), distributions of cell neighborhood correlations (middle), and odds ratios (right) are shown. (**G**) Expression of selected surface proteins within NK cells. All the statistical association tests were adjusted for age and sex. For all CNA-based association results, cells in UMAP are colored in red (expansion) or blue (depletion), and *P* value is shown as well. Error bars for odds ratios represent 95% confidence intervals.

We observed other immune cell phenotypes altered in ARIs, including expansion of CD15^+^ classical monocytes (cM) (M-0) (OR = 1.30, *P* = 0.001) in myeloid cells (Figure 4, A–C), and expansion of PAX5^lo^ naive B cells (B-6) (OR = 1.35, *P* = 0.009) uniquely in the FDR^+^ACPA^+^ ARI subgroup, the highest risk group for RA (Figure 5, A–C). To investigate the phenotype of PAX5^lo^ naive B cells, we compared the expression of activation markers that decrease with B cell activation (CD21 and CD23) (64, 65) in the largest naive cluster (B-0). Notably, PAX5^lo^ naive B cells were characterized by lower expression of CD21 and CD23 than conventional naive B cells, suggesting an activated state of PAX5^lo^ naive B cells (Figure 5D). The PAX5^lo^ naive B cluster (B-6) also expressed higher IgM protein expression levels than conventional naive B cells (B-0) (Figure 2B and Supplemental Figure 5). Within NK cell clusters, we observed an expansion of the CD56^dim^CD16^+^CD2^+^CD57^dim^ proliferating (Ki67^+^) cluster (NK-4), and a depletion of CD56^dim^CD16^+^CD2^–^CD57^–^ cells (NK-5) (Figure 5, E–G).

### ACPA status is a main driver of the cellular heterogeneity in ARIs.

**Figure 6:**
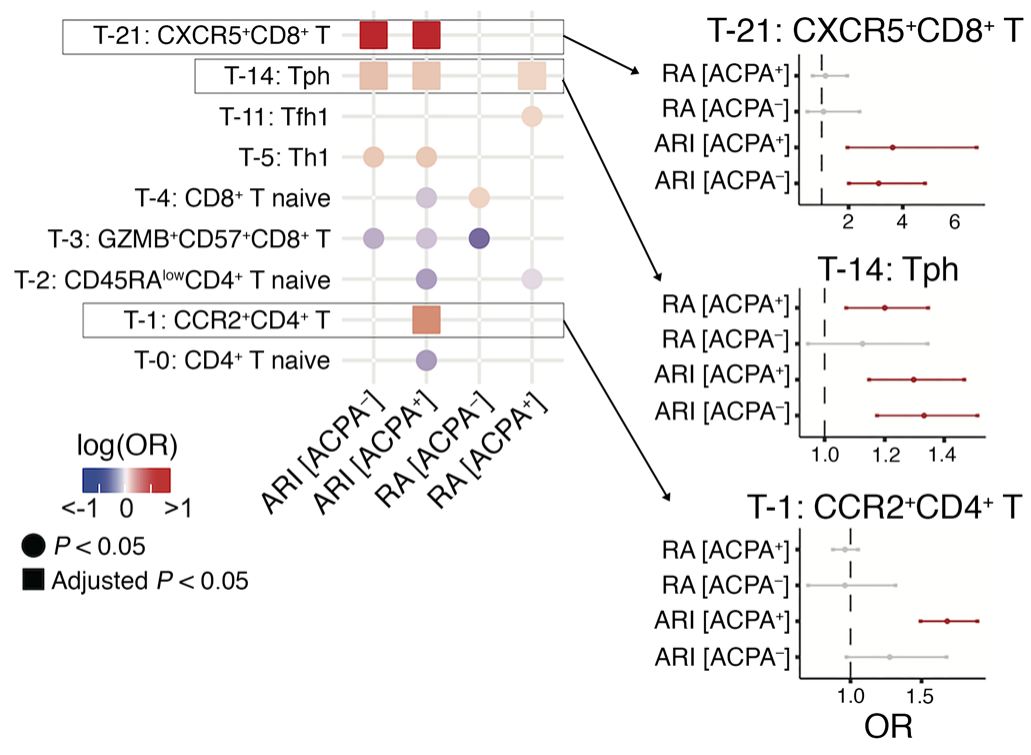
ACPA status–specific analysis reveals unique populations for different disease statuses for T cells. Heatmap shows association with each ACPA status subgroup in ARIs and RA patients (vs. controls) for each cell type. Only clusters with *P* < 0.05 are shown. Circles represent *P* < 0.05, and squares represent adjusted *P* < 0.05. Adjusted *P* values were calculated by the Benjamini-Hochberg method. Cell types are colored in red (expanded) or blue (depleted). Error bars on selected cell populations represent 95% confidence intervals. All the results in this analysis were adjusted for age and sex.

Both the presence of serum ACPAs and a family history of RA confer risk of RA (2); however, it is unclear whether these 2 risk factors are associated with similar or distinct cellular changes in the pre-RA stage. Thus, we systematically characterized enrichment levels of 79 immune cell states in the ACPA^+^ ARIs, including FDR^+^ and FDR^–^, ACPA^–^FDR^+^ ARI, ACPA^+^ RA, and ACPA^–^ RA, compared with controls. Consequently, we identified 13 cell states that exhibited either significant enrichment (OR > 1) or depletion (OR < 1) in these individual groups (adjusted *P* < 0.05), suggesting different immune signatures according to ACPA status (Supplemental Figure 10 and Supplemental Tables 4 and 5). Importantly, Tph cells (T-14), a population expanded in RA synovium, were found to be expanded in blood from both ACPA^+^ and ACPA^–^ ARI subgroups, while CCR2^+^CD4^+^ T cells (T-1) were expanded specifically in the ACPA^+^ ARI subgroup (Figure 6). In addition, the CXCR5^+^CD8^+^ T cells (T-21) were expanded in both ACPA^+^ and ACPA^–^ ARIs while not enriched in RA patients. This may suggest a pre-RA–specific immunophenotype, which warrants validation with longitudinal cohorts. In contrast, CD15^+^ cM (M-0) was expanded only in ACPA^–^ ARIs, while CD15^–^ cM (M-1) was expanded only in ACPA^+^ ARIs (Supplemental Figure 10). Further, we observed a trend of expansion of PBs (B-10) only in ACPA^+^ RA (Supplemental Figure 10). Through sensitivity analysis, we found that the FDR^–^ACPA^+^ ARI associations were concordant when comparing site-matched controls and controls from multiple clinical sites (*R* = 0.52, *P* = 2 × 10^–4^) (Supplemental Figure 11). Similarly, the FDR^+^ACPA^+^ ARI associations were significantly concordant (*R* = 0.29, *P* = 0.033) (Supplemental Figure 11). In all, these analyses suggest that these overabundant immune phenotypes (e.g., CCR2^+^CD4^+^ T cells) unique for the ACPA^+^ ARI subpopulation are maintained across multiple types of analysis.

### Single-cell transcriptomics analysis reveals Th22- and Th17-related signatures in CCR2^+^CD4^+^ T cell phenotype.

**Figure 7:**
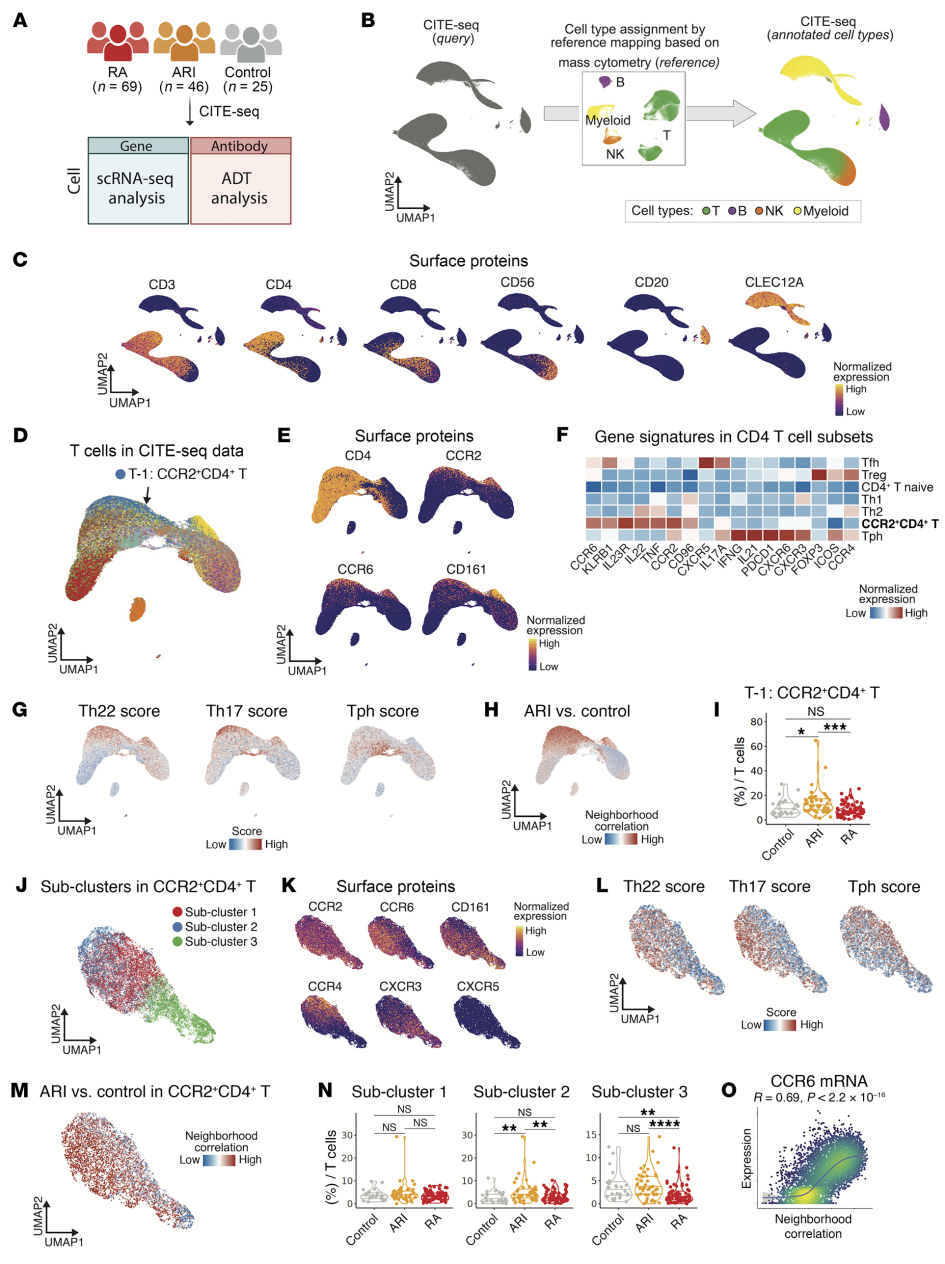
Validation using CITE-Seq data and molecular phenotype of CCR2^+^CD4^+^ T cells. (**A**) Composition and experimental design of CITE-Seq data, involving 69 participants with RA, 46 ARIs, and 25 controls. CITE-Seq includes single-cell RNA-Seq and antibody-derived tag (ADT) analysis to assess gene and protein expression. (**B**) Reference mapping assigned concordant cell clusters with mass cytometry data to CITE-Seq data. De novo cell clusters in CITE-Seq data are shown in the left UMAP plot. Through reference mapping using mass cytometry data as a reference, their cell cluster labels were transferred to the corresponding CITE-Seq clusters, effectively annotating the unidentified clusters with known cell types, as shown in the right plot. (**C**) UMAP plots of surface protein expression for key markers (CD3, CD4, CD8, CD56, CD20, CLEC12A) across PBMCs. Color intensity represents normalized expression levels of each marker, indicating presence and distribution of various cell populations. (**D**) UMAP plot of T cells from CITE-Seq data. CCR2^+^CD4^+^ T cells are labeled and colored in blue. Other colors correspond to cluster colors in Figure 2A. (**E**) UMAP plots depicting expression patterns of Th17- and Th22-related surface proteins. (**F**) Heatmap showing normalized expression levels of Th17- and Th22-related genes across helper T cell subsets. (**G**) UMAP colored by enrichment of Th22, Th17, and Tph gene signatures. (**H**) Associations of T cell neighborhoods with ARIs versus controls. For CNA-based association results, cells in UMAP are colored in red (expansion) or blue (depletion). (**I**) Distribution of cell type frequency for CCR2^+^CD4^+^ T cells. *P < 0.05, ***P < 0.001 by 2-sided Wilcoxon’s test. (**J**) UMAP plot of subclusters in CCR2^+^CD4^+^ T cells. (**K**) UMAP plots depicting expression of surface proteins for helper T cell subsets. (**L**) UMAP plots colored by enrichment of Th22, Th17, and Tph gene signatures. (**M**) Associations of CCR2^+^CD4^+^ T cell neighborhoods with ARIs versus controls. For all CNA-based association results, cells in UMAP are colored in red (expansion) or blue (depletion). (**N**) Distribution of cell type frequency for subclusters in CCR2^+^CD4^+^ T cells. **P < 0.01, ****P < 0.0001 by 2-sided Wilcoxon’s test. (**O**) Scatterplot showing correlation between ARI association obtained from CNA in **H** and mRNA expression level of *CCR6*.

We sought to validate our findings regarding reproducibility using single-cell multimodal sequencing technology. To facilitate this, we generated additional CITE-Seq data to assess gene and protein expressions in largely the same cohort of individuals. After quality control steps (Methods), we retained a total of 488,540 high-quality cells from 69 patients with RA, 46 ARIs, and 25 controls (Figure 7A and Supplemental Figure 12). This allows for a comprehensive validation of cellular phenotypes and molecular markers based on both mRNA and protein expression. Using reference mapping techniques (66), we aligned de novo cell clusters identified in the CITE-Seq data with previously established clusters from mass cytometry analyses (66) (Figure 7, B and D) and confirmed concordant expression of specific protein markers in relevant cell clusters (Figure 7, C and E) for PBMCs and T cells, respectively. Interestingly, reanalysis of markers for helper T cell subsets showed high expression levels of Th17- and Th22-associated markers, such as CCR6, IL-23R, KLRB1 (encoding CD161), IL-22, and CD96, on the CCR2^+^CD4^+^ T cells, as well as CCR2 and TNF (Figure 7, E and F). CCR2^+^CD4^+^ T cells showed the highest enrichment score for both Th22- and Th17-associated gene sets (Figure 7G). As for ARI association, we observed an enrichment of cell neighborhoods among CCR2^+^CD4^+^ T cells in cells from ARIs compared with controls, although the CNA-based *P* value was not significant (*P* = 0.587) (Figure 7H). However, there was a significant increase in the CCR2^+^CD4^+^ cell cluster frequency in ARIs compared with both controls and patients with RA (Figure 7I). We also found strong concordance in effect sizes for the ARI association (Figure 7J) and established RA association (Supplemental Figure 12G) across all T cell subsets between mass cytometry and CITE-Seq datasets. Further, correlation analyses revealed that the expression levels of Th22, Th17, and Tph gene signatures were positively correlated with the ARI association metrics from the CNA model, the association of each cellular neighborhood (Supplemental Figure 12H).

Finally, we investigated dominant signatures within CCR2^+^CD4^+^ T cells, recognizing that CCR2 is broadly expressed in various T cell subsets such as Th1, Th2, Th17, and Tph cells. To address this, we performed subclustering analysis for the CCR2^+^CD4^+^ T cells in CITE-Seq data and identified 3 subclusters. Subcluster 1 expressed Th2-related surface proteins (e.g., CCR4), subcluster 2 expressed Th17- and Th22-related surface proteins (e.g., CCR6 and CD161), and subcluster 3 expressed Th1-related surface proteins (e.g., CXCR3) (Figure 7, J and K). All subclusters shared the expression of CCR2 and the absence of CXCR5. Notably, subcluster 2 exhibited a predominance of Th17 and Th22 signatures rather than the Tph signature, as confirmed by aggregated mRNA expression analysis (Figure 7L). As for disease association, we observed a trend toward ARI association among the cell neighborhoods within CCR2^+^CD4^+^ T cell subclusters (*P* = 0.097) (Figure 7M), with a higher abundance of subcluster 2 in ARIs (Figure 7N). Among the measured mRNAs, *CCR6* emerged as the gene most correlated with ARI association metrics according to CNA (Figure 7O). These findings highlight the Th17- and Th22-related phenotypes of the CCR2^+^CD4^+^ T cells, independent from Tph cells and Th1 cells, potentially implicating them in immune dysregulation in ARIs.

## Discussion

We constructed an at-risk landscape of immune cell atlas in blood using comprehensive surface proteins of large-scale single-cell proteomics (>8,000,000 cells). Through robust computational modeling and integrative analyses, we discovered cell clusters from different immune cell compartments that are significantly altered in ARIs. Given the limited availability of pre-RA cohorts and the challenge to harmonize cross-site single-cell data, our integrative and disease association strategies can be easily generalized to maximize the power to address similar disease progression questions.

During the past decade, research on established RA has been focusing on genetics and transcriptional regulation, as well as gene and protein expression, which have uncovered biologically meaningful signatures (46, 50, 67–73). Single-cell transcriptomic and multimodal analysis have revealed high granular cell populations in the inflamed synovium to pinpoint phenotypes that characterize tissue inflammation (46, 50). Recent clinical trial studies that examine synovial heterogeneity using bulk RNA-Seq suggest that treatment response may depend on the specific immune composition in the tissue (74, 75). More importantly, translating already identified RA-relevant signatures from tissue to inform prognosis or even predict clinical RA onset in the clinic is still challenging because of the knowledge gap of immunophenotypes in the at-risk stage. Our study is among the first unbiased reports using a combination of mass cytometry and CITE-Seq to characterize the immune heterogeneity within different subsets of ARIs. We detected pathogenic cell populations and also uncovered their distinct phenotypes. For example, we identified and further defined the expansion of Th1 and Tfh1 in ARIs, which is consistent with the predominance of Th1 response over Th2 in RA (76, 77). Although Tph cells are known to be increased in RA, especially in seropositive RA (57), we report here that these cells are also overabundant in the circulation of ARIs, including not only ACPA^+^ but also, for the first time to our knowledge, ACPA^–^ ARIs. Given that the ACPA^–^ ARIs in our data are FDRs and the ACPA status can reflect the genetic background in RA (78, 79), the ACPA^−^ ARIs could include the population that will become ACPA^+^ in the future, and the expansion of Tph cells in this population may reflect this potential. Alternatively, the presence of expanded Tph cells may reflect either the genetic or the environmental influences of being an FDR of an RA patient, regardless of ACPA status.

We also found an expanded CCR2^+^CD4^+^ T cluster in ARIs, and our analysis using an external single-cell dataset further demonstrated that these CCR2^+^CD4^+^ T cells in the blood showed strong signatures of Th22 cells (e.g., CCR6^+^, IL-23R^+^, KLRB1/CD161^+^, IL-22^+^), supporting the possibility that expanded Th17/Th22 cells, which play important roles at mucosal sites, may contribute to the mucosal origin endotype of RA (24, 29). To that end, recent research showed that mono-colonization of mice with a specific strain of *Subdoligranulum didolesgii*, expanded in the feces of ARIs, led to an expansion of splenic Th17 cells, serum RA-relevant autoantibodies, and joint swelling reminiscent of early RA (25). Another study reported an increased proportion of Th17 cells specific for citrullinated cartilage intermediate layer protein in ARIs (44). However, the mechanisms by which these Th17 cells may cause RA remain unclear. Multiple large-scale bulk RNA-Seq datasets also support that *CCR2* can be expressed by Th17 cells (80–82). A recent paper demonstrated that the IL-23/IL-23R signal drives a change in chemokine receptor usage from CCR6 to CCR2 and stimulates the production of inflammatory cytokines, including TNF, in the central nervous system (83). Further, another study demonstrated that the IL-23/IL-23R axis in Th17 cells determines the onset of autoimmune arthritis, rather than its established phase, by promoting the proinflammatory activity of autoantibodies via producing IL-21 and IL-22, not IL-17 (84). IL-22 is known as the primary cytokine of Th22 cells (85), which were discovered in analogy to the Th17 subset (86). IL-22 increases the proliferation of RA synovial fibroblasts and drives their production of CCL2 (87). Moreover, Th22 cells, which infiltrate the synovial tissue in patients with active RA but not in patients with osteoarthritis, promote osteoclast differentiation through production of IL-22 (88). Research from another group indicated that the Th22-related gene expression program was associated with RA (89). Together with our findings, these studies highlight the potential mechanism of Th17 cells in driving the initial break in tolerance and disease progression in RA through dynamic phenotypic change to Th22 cells upon IL-23/IL-23R stimulation. Considering that the ligand for CCR2, CCL2, is abundantly produced from fibroblasts in inflamed synovium (50), CCR2 may help induce migration of this CCR2^+^CD4^+^ T cluster into inflamed synovium (63). Future detailed study on CCR2^+^CD4^+^ T cells is necessary to define their longitudinal phenotypes across different tissues, such as mucosal tissue, synovium, and blood.

We also found that the CXCR5^+^CD8^+^ T cell population, expressing multiple chemokine receptors (e.g., CXCR3, CX3CR1, and CCR4), was expanded in the ARIs. Previous research shows that the CXCR5-expressing follicular CD8^+^ T cells migrate into B cell follicles and are important in the response to chronic viral infection (90–97) and cancers (98–100). This cell population in our dataset is relatively small; thus further experimental validation is needed to investigate whether it is consistent with previously reported CXCR5-expressing CD8^+^ T cells. Assessment of the T cell receptor repertoire of this T cell population in the pre-RA phase using the approach in our parallel study (101) may also enhance our understanding of its origin and function.

A few studies have investigated the efficacy of disease-modifying antirheumatic drugs in the pre-RA period (19, 20, 102, 103). One critical challenge is how to precisely identify the high-risk individuals who actually need intervention, because only a subset of ARIs will develop RA in the near term, and there are not yet well-established strategies for prevention (19, 20, 102, 103). Further, because preventive studies are aimed at people who have not yet developed RA, a balance must be struck between potential adverse events and efficacy, and the use of predictive markers for treatment response may be useful (104). For example, B cell–directed depletion therapy in ARIs delays RA onset (20). Here, we identified a PAX5^lo^ naive B cell phenotype overabundant in FDR^+^ACPA^+^ ARIs. This B cell population may represent a pre-plasmablast state given that PAX5 is a key transcription factor in B cell development but is repressed during plasma cell differentiation (105, 106). Additionally, the activated naive B cells that expanded in systemic lupus erythematosus (SLE) were characterized by low expression of surface CXCR5, CD21, and CD23, in addition to high expression of CD11c and T-bet (107), suggesting that PAX5^lo^ naive B cells might be a different subpopulation compared with the one expanded in SLE. In parallel, the T cell costimulatory molecule inhibitor CTLA4-Ig in pre-RA inhibits progression to RA and decreases image-based inflammation and clinical symptoms (21, 22, 102). The CCR2^+^CD4^+^ T cells we identified and the Tph cells have the potential to be considered as predictive markers given their expansion in ACPA^+^ ARIs and highly expressed proteins for B cell help. We also found a myeloid population, CD15^+^ classical monocytes, particularly expanded in seronegative ARIs. It is not known whether any of these ARIs will develop RA with seroconversion in the future; thus, further investigating the mechanisms of these phenotypes in the natural history of RA may help delineate a more precise predictive marker.

Advances in computational integration algorithms facilitated the cross-institution, cross-tissue, and cross-disease analyses, revealing underlying shared mechanisms and pathways in immune-mediated diseases (47, 49, 55, 108–111). Most of these studies primarily integrated data on mRNA expression levels and chromatin states. Here, we present single-cell proteomics references along with single-cell multimodal transcriptomics evaluations that comprehensively incorporated ARIs and patients with established RA, which can serve as references to query immune phenotypes involved during RA progression and conversion (e.g., early or established RA, before or after treatment). Our study also includes samples at different time points from the same RA individuals, although they are not sufficient to test the changes of unique cell populations caused by treatments in this study (Supplemental Table 1). Further, our references will help clarify the blood-tissue comparison to elucidate the circulation pathways of the pathogenic immune phenotypes identified in this study and their migration mechanisms between blood and tissue. The next stages of study should aim to determine how the phenotypes characterized herein relate to other phenotypes in ARIs, including alterations in the lung mucosa (30) and gut microbiome (25), among others.

## Methods

For additional details of methods, please refer to Supplemental Methods.

### Sex as a biological variable.

We accounted for sex as a biological variable. As shown in Supplemental Table 1, there were no significant differences in the sex distribution among clinical categories. Furthermore, we adjusted for sex as a confounding factor, ensuring it was appropriately considered in our analyses, as described in other relevant sections of the article.

### Subject recruitment and clinical data collection.

The AMP RA/SLE Network constructed a cross-sectional cohort. PBMC samples from RA and controls were collected from 11 clinical sites across the United States and 2 sites in the United Kingdom. RA individuals were diagnosed based on the 2010 American College of Rheumatology/European Alliance of Associations for Rheumatology criteria (112). Healthy controls from 3 clinical sites were tested for ACPAs and rheumatoid factor (RF), and they were negative. The original samples from ARIs without inflammatory arthritis seen in rheumatology clinics were collected from the University of Colorado Anschutz Medical Campus. Demographics and clinical data were collected, and measurements of laboratory data were performed at the baseline visit. Data collected include age, sex, race, RF or ACPA status, RA treatments, and tender and swollen joint counts. For ARIs, we defined ACPA-positive as anti-CCP3 and/or anti-CCP3.1 titer ≥ 20 units; for ACPA-negative classification, individuals were negative for both of these assays. For established RA, we defined ACPA-positive as anti-CCP1, anti-CCP2, and/or anti-CCP3 titer more than the upper limit of normal range of each clinical site based on local laboratory standards and reporting. Erythrocyte sedimentation rate and C-reactive protein were measured for established RA patients using commercial assays in each institution’s clinical laboratory. Disease activity for each RA patient was calculated using the Clinical Disease Activity Index (113). For validation, samples from ARIs and controls were collected from the same 2 clinical sites, the University of Colorado Anschutz Medical Campus and Brigham and Women’s Hospital; and the ARIs from both sites were defined and characterized using the same classification strategies, family history and positivity for ACPAs, for validation.

### Statistics.

The statistical tests performed are indicated in the figure legends and as follows. For the analysis of our data, the Mann-Whitney *U* test was used to compare continuous variables between 2 groups, and the Kruskal-Wallis test for comparisons involving more than 2 groups. Spearman’s rank correlation was used to examine the relationship between 2 continuous variables. Categorical variables were compared using Fisher’s exact test or the χ^2^ test, chosen based on the sample sizes of the categories involved. We corrected for multiple comparisons using the Benjamini-Hochberg procedure to control the false discovery rate. An adjusted *P* value less than 0.05 was considered significant. For cell frequency plots, additional corrections for multiple comparisons were not applied, as these were generated based on cell types pre-prioritized by MASC and CNA analyses, which already included log-likelihood ratio tests with adjusted *P* values and significance testing through permutation analysis. Missing data were handled by exclusion of incomplete cases from the respective analyses to ensure the integrity of the results.

### Study approval.

The study was performed in accordance with protocols approved by the institutional review board at the University of Colorado Anschutz Medical Campus. Written informed consent was received prior to participation. The design and conduct of this study fully complied with all relevant regulations regarding the use of human study participants and adhered to the ethical principles outlined in the Declaration of Helsinki.

### Data and code availability.

The results published here are in whole or in part based on data obtained from the ARK Portal (http://arkportal.synapse.org). The AMP RA/SLE Network data used for this publication are available at https://doi.org/10.7303/syn64425850.2 and https://arkportal.synapse.org/Explore/Datasets/DetailsPage?id=64425850 The ARK Portal hosts data generated by a network of research teams working collaboratively to deepen the understanding of arthritis and autoimmune and related diseases. It was established by the National Institute of Arthritis and Musculoskeletal and Skin Diseases (NIAMS) and includes data from the AMP RA/SLE program. Values for all data points in graphs are reported in the Supporting Data Values file. All source code to reproduce the analyses is available on GitHub (https://github.com/fanzhanglab/AtRiskRA_SingleCell/). Supplemental material for this paper is available online.

## Author contributions

KDD, VMH, JA Seifert, MLF, and JMN recruited patients, obtained samples, and curated clinical data for the SERA cohort study. JAJ, MBB, SR, WA, SLB, VPB, S Goodman, LTD, GSF, HP, JMB, LBH, AF, CP, JHA, LM, and JMG contributed to the procurement and processing of samples and design of the AMP RA/SLE study. KDD, VMH, and JA Sparks recruited and obtained samples for validation study. JL, JK, AG, KH, JFP, EM, and BH designed and implemented the sample preparation, cell sorting, and mass cytometry data generation pipeline. STD, MGG, S Gurajala, NH, and CMC contributed to the CITE-Seq data generation. JI led the computational and statistical analyses with support from TG, AG, and YC. For result interpretation, JI, AH, DAR, FZ, and AHJ provided disease immunology inputs. JI and FZ wrote the initial draft. JI, FZ, DAR, KDD, and VMH edited the manuscript. FZ supervised the research with cosupervision provided by DAR, JAL, KDD, and VMH. All authors participated in editing the final manuscript.

## Supplementary Material

Supplemental data

Supplemental table 1

Supplemental table 2

Supplemental table 3

Supplemental table 4

Supplemental table 5

Supplemental table 6

Supplemental table 7

Supporting data values

## Version 1 03/17/2025

Electronic publication
