# Supplementary material for: Deep immunophenotyping reveals circulating activated lymphocytes in individuals at risk for rheumatoid arthritis
Source: J Clin Invest. 2025 Mar 17;135(6):e185217. doi: 10.1172/JCI185217 (PMC11910230; doi:10.1172/JCI185217)
Supplement: Supplemental data [file jci-135-185217-s113.pdf]

Collection of samples and processing

We utilized PBMCs from individuals at-risk for RA (ARI, n=52) and established RA (n=67) who were enrolled in the AMP RA/SLE Network. ARI were sub-categorized based on their family history and/or the positivity of ACPA into FDR+ACPA- (n=23), FDR-ACPA+ (n=9), and FDR+ACPA+ (n=20). Similarly, RA patients were categorized into ACPA+ and ACPA-. For comparison, we collected PBMCs from healthy individuals as controls (n=48). Samples were shipped to the central AMP RA/SLE Biorepository, Oklahoma Medical Research Foundation Biorepository, until sample collection was complete. All the collected PBMC samples (n=167) with other consortium samples (Systemic lupus erythematosus (SLE), n=140) were randomly distributed based on disease status, clinical site, and sex into 23 technical batches to minimize effects from site differences and other demographics.

We then applied computational integrative and association algorithms to identify unique co-varying phenotypical changes across different preclinical and clinical individual groups. We applied an optimized downsampling strategy to analyze all mononuclear cells as well as specific immune cell lineages for computational efficiency. We next performed a sensitivity analysis by changing the downsampling proportions and confirmed that the immune cell clusters detected by different downsampling parameters are stable (**Extended Data Fig. 1**). In total, we analyzed 1,640,747 cells for all mononuclear cells analysis (167 individuals), and 2,196,578 T cells (163 individuals), 1,886,084 myeloid cells (161 individuals), 1,918,711 B cells (167 individuals), and 2,008,997 NK cells (160 individuals) for each cell type analysis. To correct the technical batch effect and inter-individual variation, we applied a single-cell batch effect correction algorithm (1) and quantified the improvement of mixture levels across technical batches, clinical sites, and individual samples after correction (1, 2). After batch effect correction, the degree of mixing levels across batches, race, and sites was significantly increased compared to before correction (**Extended Data Fig. 2**). For accurate integration, we confirmed that the mixing levels for cell

type, measured by LISI (Local Inverse Simpson's Index)(1, 3), as equal to 1, reflecting a correct separation of unique cell types throughout the integrative embedding. One individual with established RA whose baseline sample was not available was not included in the comparative analyses, which specifically required baseline samples (e.g., RA vs. Control comparisons at baseline).

Mass cytometry antibody staining and quality control

All PBMC samples from 167 individuals (established RA (n=67), ARI (n=52) and controls (n=48)) were thawed in a 37 °C water bath for 3 minutes and then mixed with 37 °C thawing media containing: RPMI Medium 1640 (Life Technologies #11875-085) supplemented with 5% heat-inactivated fetal bovine serum (Life Technologies #16000044), 1 mM GlutaMAX (Life Technologies #35050079), antibiotic-antimycotic (Life Technologies #15240062), 2 mM MEM non-essential amino acids (Life Technologies #11140050), 10 mM HEPES (Life Technologies #15630080), 2.5×10^{-5} M 2-mercaptoethanol (Sigma-Aldrich #M3148), 20 units/mL sodium heparin (Sigma-Aldrich #H3393), and 25 units/mL benzonase nuclease (Sigma-Aldrich #E1014). 100 µL aliquots of each sample post-thaw were mixed with PBS (Life Technologies #10010023) at a 1:1 ratio to be counted by flow cytometry. Between $0.5 – 1.0 \times 10^6$ cells were used for each sample. All samples were transferred to a polypropylene plate (Corning #3365) to be stained at room temperature for the rest of the experiment.

The samples were spun down and aspirated. Rhodium viability staining reagent (Standard BioTools #201103B) was diluted at 1:1000 and added for five minutes. 16% stock paraformaldehyde (Fisher Scientific #O4042-500) was diluted to 0.4% in PBS and added to the samples for five minutes. After centrifugation and aspiration, Human TruStain FcX Fc receptor blocking reagent (BioLegend #422302) was used at a 1:100 dilution in cell staining buffer (CSB) (PBS with 2.5 g bovine serum albumin [Sigma Aldrich #A3059] and 100 mg of sodium azide

[Sigma Aldrich #71289]) for 10 minutes followed by incubation with conjugated surface antibodies (each marker was used at a 1:100 dilution in CSB, unless stated otherwise) for 30 minutes. All antibodies were prepared and validated by the Harvard Medical Area CyTOF Antibody Resource and Core (Boston, MA).

After centrifugation, samples were resuspended with culture media. 16% stock paraformaldehyde (Fisher Scientific #O4042-500) dissolved in PBS was used at a final concentration of 4% for 10 minutes to fix the samples before permeabilization with the FoxP3/Transcription Factor Staining Buffer Set (ThermoFisher Scientific #00-5523-00). The samples were incubated with SCN-EDTA coupled palladium barcoding reagents for 15 minutes followed by incubation with Heparin (Sigma-Aldrich #H3149-100KU) diluted 1:10 in PBS. Samples were combined and filtered in a polypropylene tube fitted with a 40μm filter cap. Conjugated intracellular antibodies were added into each tube and incubated for 30 minutes. Cells were then fixed with 4% paraformaldehyde for 10 minutes.

To identify single cell events, DNA was labeled for 20 minutes with an 18.75 μM iridium intercalator solution (Standard BioTools #201192B). Samples were subsequently washed and reconstituted in Cell Acquisition Solution (CAS) (Standard BioTools #201240) in the presence of EQ Four Element Calibration beads (Standard BioTools #201078) at a final concentration of $1x10^6$ cells/mL. Samples were acquired on a Helios CyTOF Mass Cytometer (Standard BioTools). The raw FCS files were normalized to reduce signal deviation between samples over the course of multi-day batch acquisitions, utilizing the bead standard normalization method established by Fink et al (4). The normalized files were then compensated with a panel specific spillover matrix to subtract cross-contaminating signals, utilizing the CyTOF based compensation method established by Chevrier et al (5). These compensated files were then deconvoluted into individual sample files using a single cell based debarcoding algorithm

established by Zunder et al (6). Pre-analysis of CyTOF staining data included a Gaussian gating strategy (7), gating on singlet cells by residual versus DNA staining, gating on bead-negative cell events, and gating on all live cells (Rhodium-negative).

Downsampling cells for all mononuclear cells, T, and myeloid panels

T cells and myeloid cells consist of a large proportion of peripheral blood. In order to save time and computational resources for downstream analysis without missing important cell states, we downsampled cells by randomly selecting cells according to individuals for analyses for all mononuclear cells, T cells, and myeloid cells as follows;

1. If 10% of total cells > 10,000, we will keep 10% of total cells
2. If 10,000 > 10% of total cells, we will keep 10,000 cells
3. If 10,000 > total cells, we will keep total cells without downsampling

For sensitivity analysis, we performed consistent clustering analysis and obtained biological cell clusters according to the proportions of downsampling (0.1%, 1%, 10%, 20%, 30%, 40%, 50%, 60%, 70%, 80%, 90%, and 100%).

Protein expression normalization and dimensionality reduction

To minimize the effect of background on the measured signal, we normalized expression data by ArcSinh transformation of data using the cytofAsinh function in cytofkit R package with cofactor = 5 for each cell type. For dimensionality reduction, we then used truncated principal component analysis (PCA) as implemented in the prcomp_irlba function from the irlba R package and calculated 20 principal components (PCs) based on the normalized mass cytometry data. During PCA, we used the most highly variable proteins by removing 10% lowest variable proteins among cells because they are uninformative. We further corrected batch effects and sample heterogeneity simultaneously with the HarmonyMatrix function from the

harmony R package to account for covariates. We next projected the cells into two dimensions with UMAP (8, 9) with default parameters.

Graph-based clustering, differential protein expression, and cell type annotation

After batch correction, we constructed shared nearest neighbor graphs derived from the top 20 PCs and applied graph-based Louvain clustering (10) at various resolution levels (0.3, 0.5, 0.7, 1.0). We selected optimized resolution values for each cell type (0.7 for T cells, 0.3 for NK cells, 0.3 for myeloid cells, 0.5 for B cells) based on silhouette width and manual check of expression of key proteins in each cluster to gain the biological interpretations that made the most sense. Unreliable clusters less than 30 cells in total were removed. In the end, we identified 26 T cell clusters (2,196,578 cells, 163 individuals), 20 B cell clusters (1,918,711 cells, 167 individuals), 17 NK clusters (2,008,997 cells, 160 individuals), 16 myeloid clusters (1,886,084 cells, 161 individuals), for a total of 79 clusters. We allocated cluster numbers based on cluster size. For each major cell type, we identified differentially expressed surface proteins by comparing cells from one cluster with all the other cells using wilcoxauc function in presto R package. We tested all proteins that were measured in each cell type. We present cluster-specific marker proteins and relative statistics in **Supplementary Table 3**. We then annotated identified clusters based on differentially expressed markers and relevant literature showing their biological functions in each cell type.

Identification of cell populations that are significantly associated with specific clinical subgroups

We evaluated whether at-risk or RA are associated with changes in the relative abundances of cell states within all mononuclear cells (coarse) and major cell type-specific manner (fine-grained). For each cell type, we applied multiple computational strategies, 1) cluster-based approach utilizing mixed effect model, Mixed-effects Association testing for Single Cells

(MASC)(11), and 2) cluster-free based approach which identifies dominant co-vary cell neighborhoods in cell type abundance across samples in one clinical group compared to the other, covarying neighborhood analysis (CNA)(12). MASC is a statistical association strategy that uses single-cell logistic mixed-effect modeling to test individual cellular populations for their association by predicting the subset membership of each cell based on fixed effects and random effects. In MASC, a null model where the subset membership of every single cell is estimated by fixed and random effects without considering the case-control status of the samples was assumed. We then measured the improvement in model fit when a fixed effect term for the case-control status of the sample was included with a likelihood ratio test. This framework allowed us to evaluate the significance and effect size of the case-control association for each cluster while controlling for inter-individual and technical variability. In our analyses, we performed MASC using the MASC() R function as follows:

$$Null\ model: log\left[\frac{Y_{i,j}}{1-Y_{i,j}}\right] = \theta_j + \beta_{age}X_{i,k} + \beta_{sex}X_{i,k} + (\phi_i|k)$$

$$Full\ model: log\left[\frac{Y_{i,j}}{1-Y_{i,j}}\right] = \theta_j + \beta_{age}X_{i,k} + \beta_{sex}X_{i,k} + (\phi_i|k) + \beta_{case}X_{i,k}$$

Here, $Y_{i,j}$ is the odds of cell *i* belonging to cluster *j* (major cell types for all mononuclear cells analysis and fine-grained cell types for each cell type analysis, respectively), θ_j is the intercept for cluster *j*, β_{age} and β_{sex} indicate the fixed-effect of age and sex for cell *i* from k^{th} sample, respectively; (ϕ_i |*k*) is the random effect for cell *i* from k^{th} sample, β_{case} indicates the effect of kth sample's case-control status. We presented our results from MASC by odds ratio with an error bar indicating 95% confidence intervals for each cluster. The statistics are summarized in **Supplementary Table 4**.

It is noted that, for clusters with small cell numbers, statistics of MASC tend to have a wide range of confidence intervals and are unreliable, making it necessary to use the cluster-free

method such as CNA (12) in combination. We use CNA to define small cell neighborhoods in the batch-corrected harmonized low-dimensional embeddings and calculate that fractional abundance of cells from each sample in each neighborhood in a neighborhood abundance matrix (NAM). By decomposing the NAM with principal component analysis (PCA), CNA defines NAM-PCs within each cell type that capture axes of heterogeneity defined by groups of neighborhoods whose abundances vary in a coordinated manner. Next, we use CNA to perform two tests: associations between ARI vs control, and RA vs control, respectively. In practice, we used the association() function in the rcna R package with default parameters, while controlling for the "age" and "sex" as covariates. As CNA utilizes a permutation test, we obtained a significant association based on a global permutation $p < 0.05$. For visualization of local associations, we indicate the particular neighborhoods driving a global significant association. In the violin plots and UMAP plots, we colored neighborhood correlations, with red and blue indicating a positive and negative correlation, respectively. To highlight important cell neighborhoods from important cell states, we put transparent parameters according to the absolute value of correlation for each cell (from 0 [completely transparent] to 1 [no transparent]). The statistics of CNA results are in **Supplementary Table 5**.

Reference mapping of independent mass cytometry T cells to the original T cell reference

We analyzed independent mass cytometry data obtained from blood of ARI (n=57), RA (n=20), and controls (n=23) enrolled from two clinical sites (University of Colorado and Brigham Women's Hospital). Samples were shipped to the same central biorepository site until sample collection was complete. They were then transited to the central pipeline site, the same lab with the original sample processing, where samples were thawed and processed in 5 batches. After removing beads and dead cells by DNA gating, we gated T cells by CD3+CD20-CD56-CD14- and downsampled in the same way as the original T cell panel. To validate our findings in the original data, we then projected 1,022,630 T cells to the original T cell reference using the

mapQuery() function based on 29 common proteins from the Symphony package. For reference building from the Harmony objects, we used the buildReferenceFromHarmonyObj() function. We predicted cell states for the query cells based on the 30 nearest cell neighbors using the knnPredict() function with k=30.

Single-cell CITE-seq antibody staining, RNA library preparation, and sequencing

PBMC samples suspended in Cryostor CS-10 and stored in liquid nitrogen were transferred on dry ice to the lab and thawed in batches of 4 at a time (up to 16 samples total) in a 37 degrees C water bath with constant swirling until ice disappeared (~1.75 min). Each sample was diluted in thawing media containing RPMI 1640 without glutamine (Gibco) supplemented with 0.5% BSA (Miltenyi Biotec), 1X Glutamax (Gibco), and 10 mM HEPES (Corning), then filtered through a 40 um strainer (pluriSelect). The filter was rinsed with an additional thawing buffer to dilute the cryopreservation media. Processing continued in batches of 16. Cells were pelleted by centrifugation (350g) and incubated for 20 minutes on ice with a Fc blocking reagent (Miltenyi Biotec) and a cocktail of fluorescent antibodies (BD Biosciences) targeting CD15 (clone: W6D3; conjugated to AF700) and CD45 (for each sample; clone:HI30; conjugated to one of the following: BB515, PE, PE/Cy7, BUV395) in autoMACS Running Buffer (Miltenyi Biotec). The samples were then washed and counted using a Cellometer Counter (Nexcelom). Pools of 4 samples were then created containing 150,000 live cells from each sample such that the CD45 fluorochrome was unique for each sample with the pool (600,000 live cells per pool). For oligo barcode-tagged surface protein detection, each pool was incubated with Totalseq-A Human Universal Cocktail V1.0 (Biolegend; 25% of manufacturer’s recommendation) prepared in Cell Staining Buffer (Biolegend) for 30 minutes at 4 degrees C in a total volume of 50 uL according to manufacturer’s instructions. Following incubation, pools were washed twice, resuspended in autoMACS Running Buffer containing 1 ug/mL DAPI (Biolegend), and filtered through a 35 um strainer. Equal numbers of live CD15 negative cells from each sample were then FACS sorted

(BD FACSAria) using the CD45 fluorochrome to distinguish the individual samples in each pool (15,000 cells per sample) into loading media containing RPMI 1640 without glutamine (Gibco) supplemented with 0.04% BSA, 1X Glutamax, and 10mM HEPES. After sorting, all 16 samples in the batch were pooled in one tube and 32,000 cells were loaded in each of 3 Chromium chips (10x Genomics) to generate single cell RNAseq and surface protein libraries using manufacturer's protocols. Completed RNA and ADT libraries were pooled at a 3 RNA:1 ADT molar ratio (75% RNA:25% ADT). Two "master" pools were then made, each containing 19 libraries (RNA+ADT), for 38 total libraries, and sequenced across 3 individual S4 flow cells on a Novaseq (Illumina) to a 5,092 reads per cell.

Single-cell CITE-seq gene expression and protein expression quantification

mRNA and antibody-derived tag (ADT) unique molecular identifier (UMI) counts were quantified using Cell Ranger v3.1.0. Raw BCL files were demultiplexed using cellranger mkfastq with default parameters to generate FASTQ files. These FASTQ files were then aligned to the GRCh38 human reference genome using Cell Ranger v3.1.0, with gene and ADT reads quantified simultaneously using cellranger count.

Quality control of single-cell CITE-seq data

Analyses for quality control, normalization, and scaling were performed following the steps outlined in Zhang., et al (13). Cells identified as doublets by scDblFinder (14) and expressing fewer than 500 genes or containing more than 20% of their total UMIs mapping to mitochondrial genes were removed, resulting in 502,799 cells. Sample-level QC was then performed, removing samples with a small number of cells (< 300). The final dataset contained 488,540 cells from 140 samples for downstream analysis. mRNA features were normalized, selected, and scaled both globally and by cell type. Global normalization involved log transformation and scaling by total UMIs per cell, followed by selection of the top 2,000 most highly variable genes

per sample based on a variance stabilizing transformation. These genes were then pooled across all samples for a cell type, and z-score scaling was applied. Cell type-specific normalization and scaling followed the same steps but were performed only on cells of each given cell type. Protein features were normalized using centered-log ratio (CLR) transformation and corrected for antibody background staining using a Gaussian mixture model. Cell type-specific protein normalization was performed in the same manner, with additional scaling steps for each cell type.

For global analysis and cell-type-specific analysis, a multi-modal dimensionality reduction strategy was used to integrate mRNA and surface protein expression. Canonical correlation analysis (CCA) was performed on scaled mRNA and protein data, followed by selection of the top 20 canonical variates, batch effect correction with Harmony, and projection into two dimensions with UMAP. To integrate and compare CITE-seq data and two mass cytometry datasets (original and validation), we employed a reference mapping approach using the StabMap (15). For the CITE-seq dataset, we selected the surface proteins corresponding to the genes present in the mass cytometry datasets. Similarly, for the mass cytometry datasets (from our study and an external dataset), we selected the surface proteins with corresponding genes in the CITE-seq dataset. The preprocessed datasets were used as input, specifying the original mass cytometry dataset as the reference and the CITE-seq as a query dataset. This step generated a low-dimensional embedding of the cells from all datasets, with the query datasets aligned to the reference dataset. To assign cell type labels to the query datasets, we trained a k-nearest neighbors (k-NN) classifier on the reference dataset using the *knn* function of class R package (16). The k-NN classifier was then used to predict cell type labels for the cells in the query datasets based on their proximity to the annotated cells in the reference dataset. The k-NN classifier was run with k = 5 and the probability of each cell type assignment was calculated.

Cell type-specific reference mapping followed the same steps but were performed only on cells of each given cell type.

The signature scores for Th22, Th17, and Tph cells were calculated using the addmodulescore function from the Seurat R package. The gene signature lists were derived from previous studies (17–19).

References

1. Korsunsky I, et al. Fast, sensitive and accurate integration of single-cell data with Harmony. *Nat Methods*. 2019;16(12):1289–1296.

2. Zhang F, et al. IFN-γ and TNF-α drive a CXCL10+ CCL2+ macrophage phenotype expanded in severe COVID-19 lungs and inflammatory diseases with tissue inflammation. *Genome Med*. 2021;13(1):64.

3. Tran HTN, et al. A benchmark of batch-effect correction methods for single-cell RNA sequencing data. *Genome Biol*. 2020;21(1):12.

4. Finck R, et al. Normalization of mass cytometry data with bead standards. *Cytometry A*. 2013;83(5):483–494.

5. Chevrier S, et al. Compensation of Signal Spillover in Suspension and Imaging Mass Cytometry. *Cell Syst*. 2018;6(5):612–620.e5.

6. Zunder ER, et al. Palladium-based mass tag cell barcoding with a doublet-filtering scheme and single-cell deconvolution algorithm. *Nat Protoc*. 2015;10(2):316–333.

7. Bagwell CB, et al. Automated Data Cleanup for Mass Cytometry. *Cytometry A*. 2020;97(2):184–198.

8. McInnes L, Healy J, Melville J. UMAP: Uniform Manifold Approximation and Projection for Dimension Reduction. *arXiv [statML]*. 2018. http://arxiv.org/abs/1802.03426.

9. Becht E, et al. Dimensionality reduction for visualizing single-cell data using UMAP. *Nat Biotechnol*. [published online ahead of print: December 3, 2018]. https://doi.org/10.1038/nbt.4314.

10. Blondel VD, et al. Fast unfolding of communities in large networks. *J Stat Mech*. 2008;2008(10):P10008.

11. Fonseka CY, et al. Mixed-effects association of single cells identifies an expanded effector CD4 T cell subset in rheumatoid arthritis. *Sci Transl Med*. 2018;10(463). https://doi.org/10.1126/scitranslmed.aaq0305.

12. Reshef YA, et al. Co-varying neighborhood analysis identifies cell populations associated with phenotypes of interest from single-cell transcriptomics. *Nat Biotechnol*. 2022;40(3):355–363.

13. Zhang F, et al. Deconstruction of rheumatoid arthritis synovium defines inflammatory subtypes. *Nature*. 2023;623(7987):616–624.

14. Germain P-L, et al. Doublet identification in single-cell sequencing data using. *F1000Res*. 2021;10:979.

15. Ghazanfar S, Guibentif C, Marioni JC. Stabilized mosaic single-cell data integration using unshared features. *Nat Biotechnol*. 2023;42(2):284–292.

16. Modern Applied Statistics with S, 4th ed [Internet]. https://www.stats.ox.ac.uk/pub/MASS4/. Accessed May 13, 2024.

17. Law C, et al. Interferon subverts an AHR–JUN axis to promote CXCL13+ T cells in lupus. *Nature*. 2024;631(8022):857–866.

18. Zhang F, et al. Defining inflammatory cell states in rheumatoid arthritis joint synovial tissues by integrating single-cell transcriptomics and mass cytometry. *Nat Immunol*. 2019;20(7):928–942.

19. Höllbacher B, et al. Transcriptomic Profiling of Human Effector and Regulatory T Cell Subsets Identifies Predictive Population Signatures. *Immunohorizons*. 2020;4(10):585–596.

Extended Data Figures

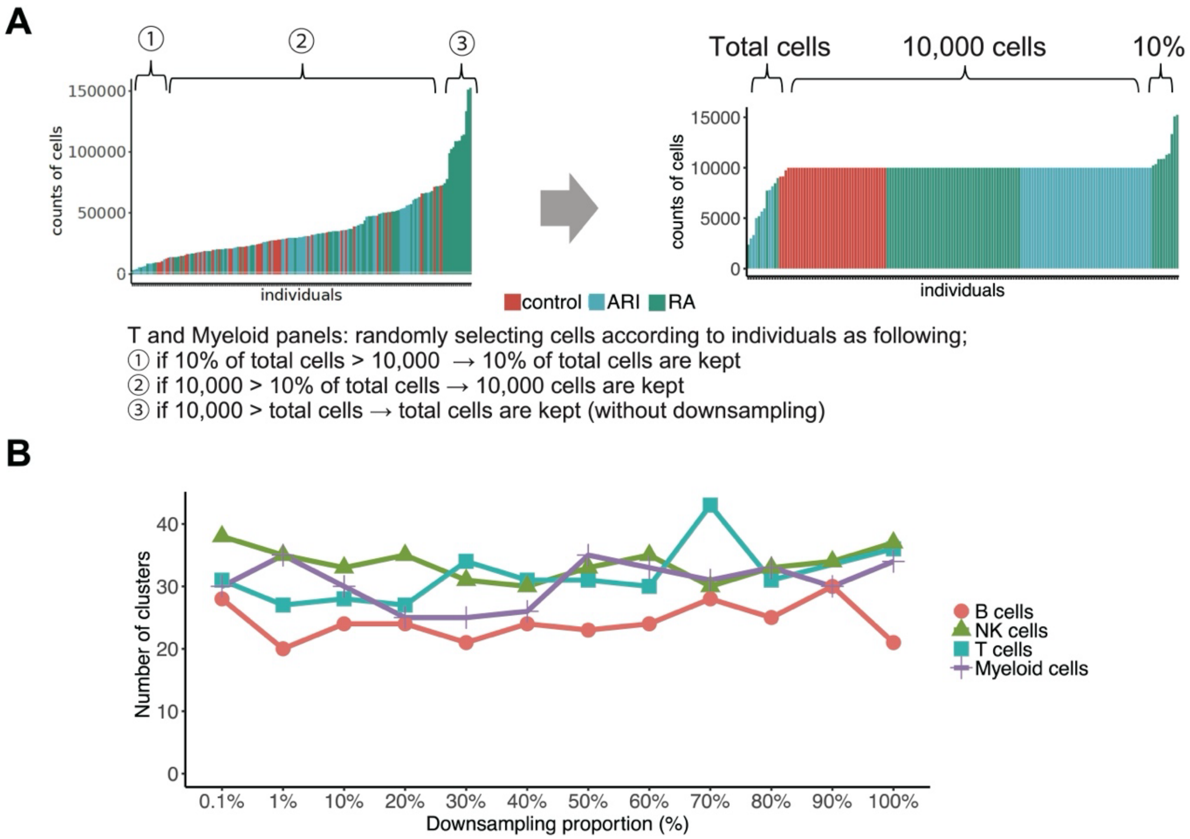

Extended Data Fig. 1: Downsampling strategy for large-scale mass cytometry dataset. A. Optimized downsampling schema developed for large-scale mass cytometry dataset to efficiently conduct downstream analysis without losing robustness, **B**. Sensitivity analysis for downsampling strategy. X-axis represents the proportion of downsampling cells. Y-axis represents the number of identified biologically meaningful cell clusters in each cell type using graph-based clustering.

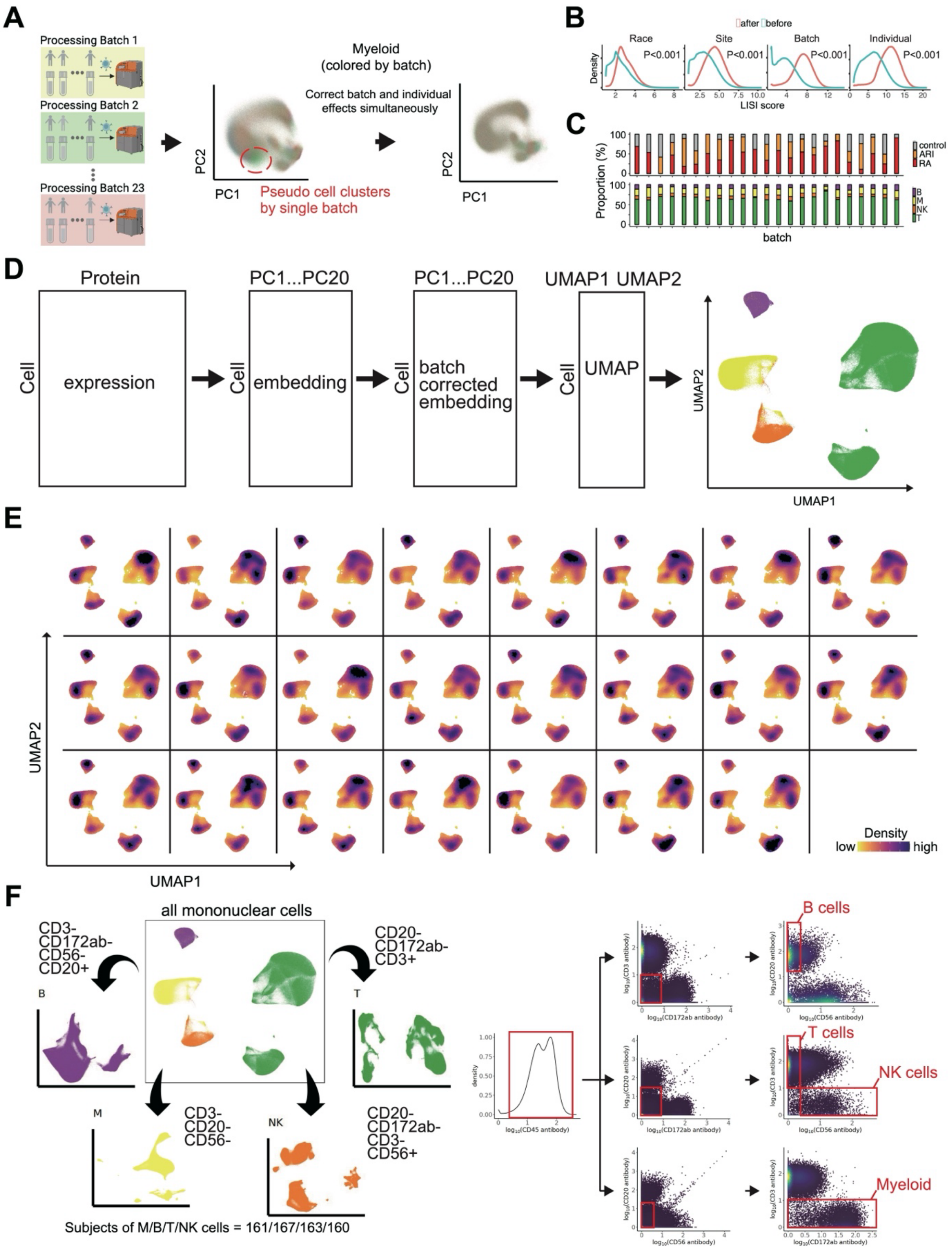

Extended Data Fig. 2: Analytical pipeline applied to large-scale mass cytometry data. A. Representative example of batch effect correction using myeloid panel. **B.** LISI scores in myeloid panel to measure mixture levels on race, clinical site, batch, and samples. After batch

effect correction, the mixture level of clinical sites (median LISI = 4.45), technical batches (median LISI = 7.16) are significantly reduced compared to before correction (median LISI = 4.31 for clinical sites, median LISI = 5.53 for technical batchers (Wilcoxon test $p < 0.01$), **C**. Distribution of samples (top) and cell types (bottom) by batch, **D**. Analytical pipeline from expression data to cell embeddings in low-dimensional space using dimensionality reduction, **E**. Density plot using all mononuclear cells by batch. Cells from different batches but the same cell types are clustered together, **F**. Gating strategy for mass cytometry data to determine selected immune cell populations.

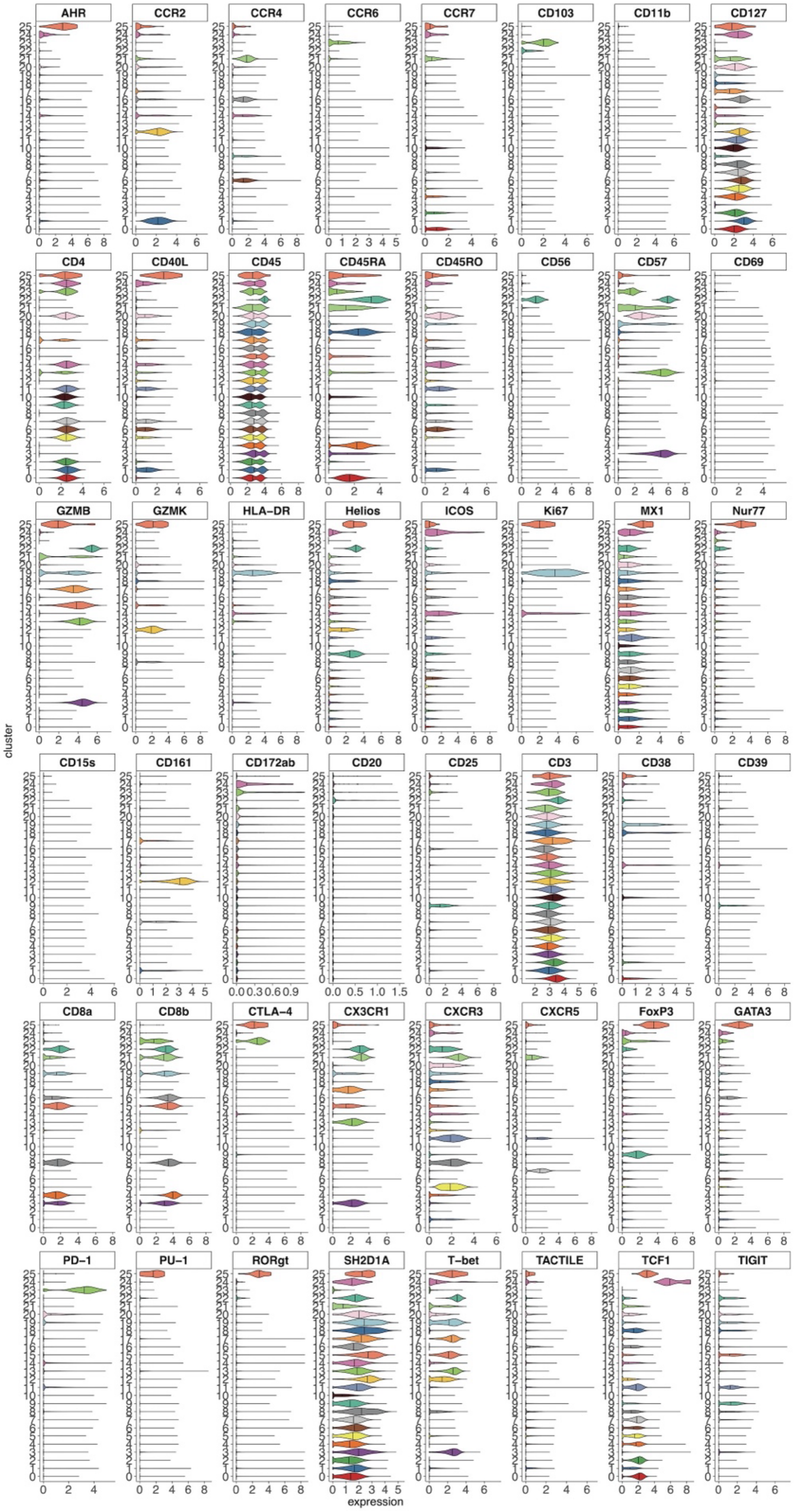

Extended Data Fig. 3: Expression of measured proteins in T cell panel.

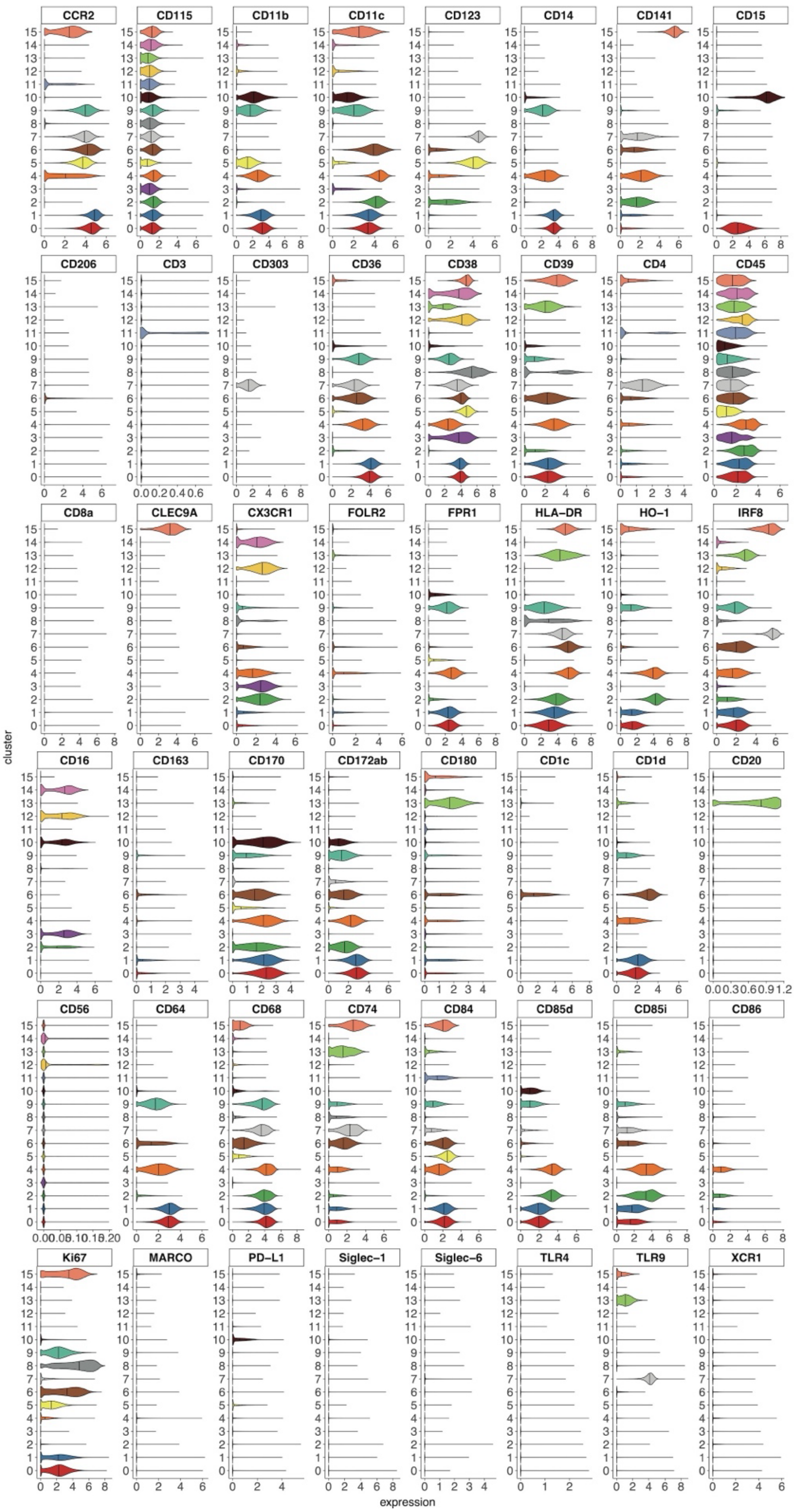

Extended Data Fig. 4: Expression of measured proteins in myeloid cell panel.

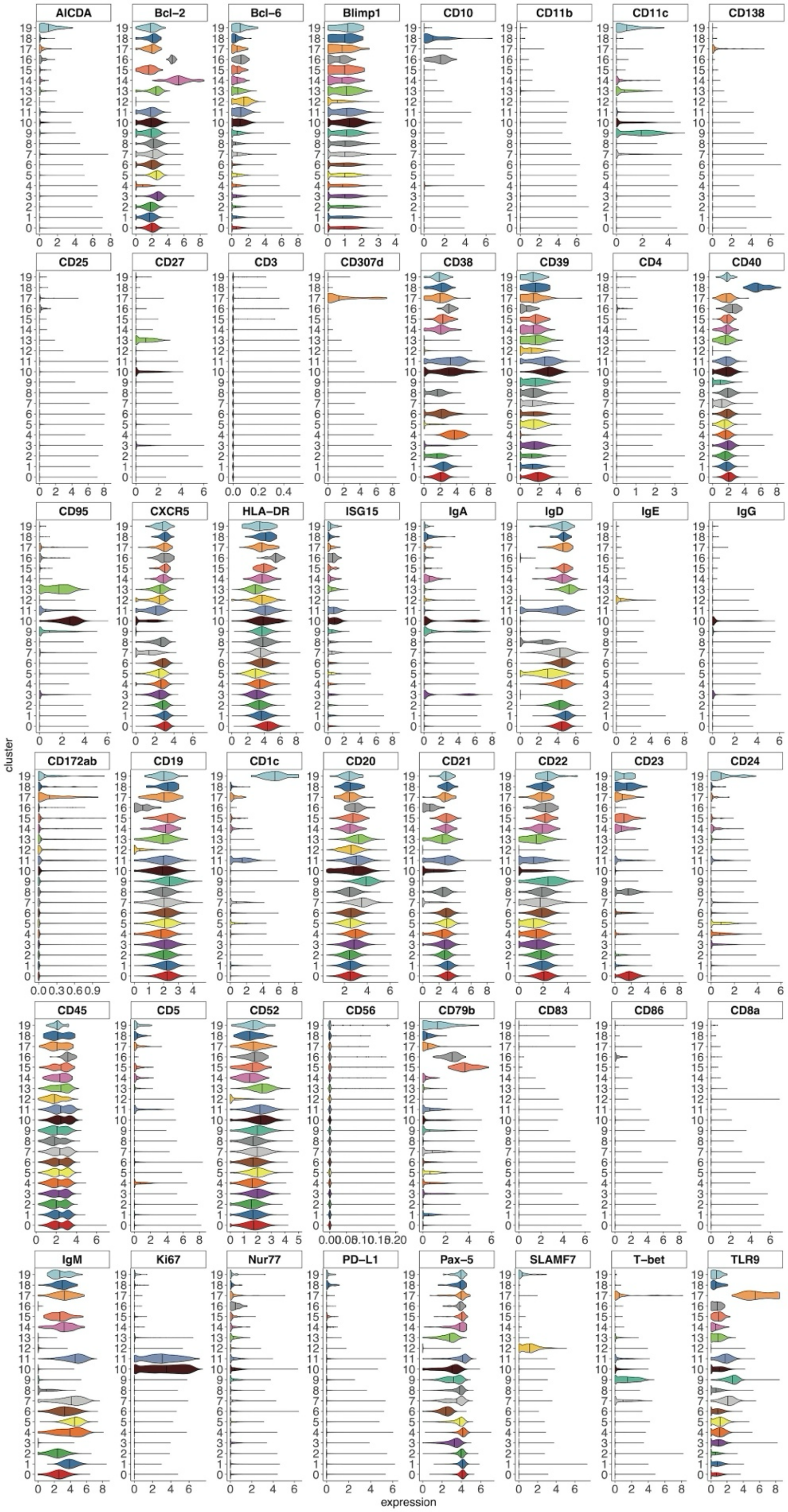

Extended Data Fig. 5: Expression of measured proteins in B cell panel.

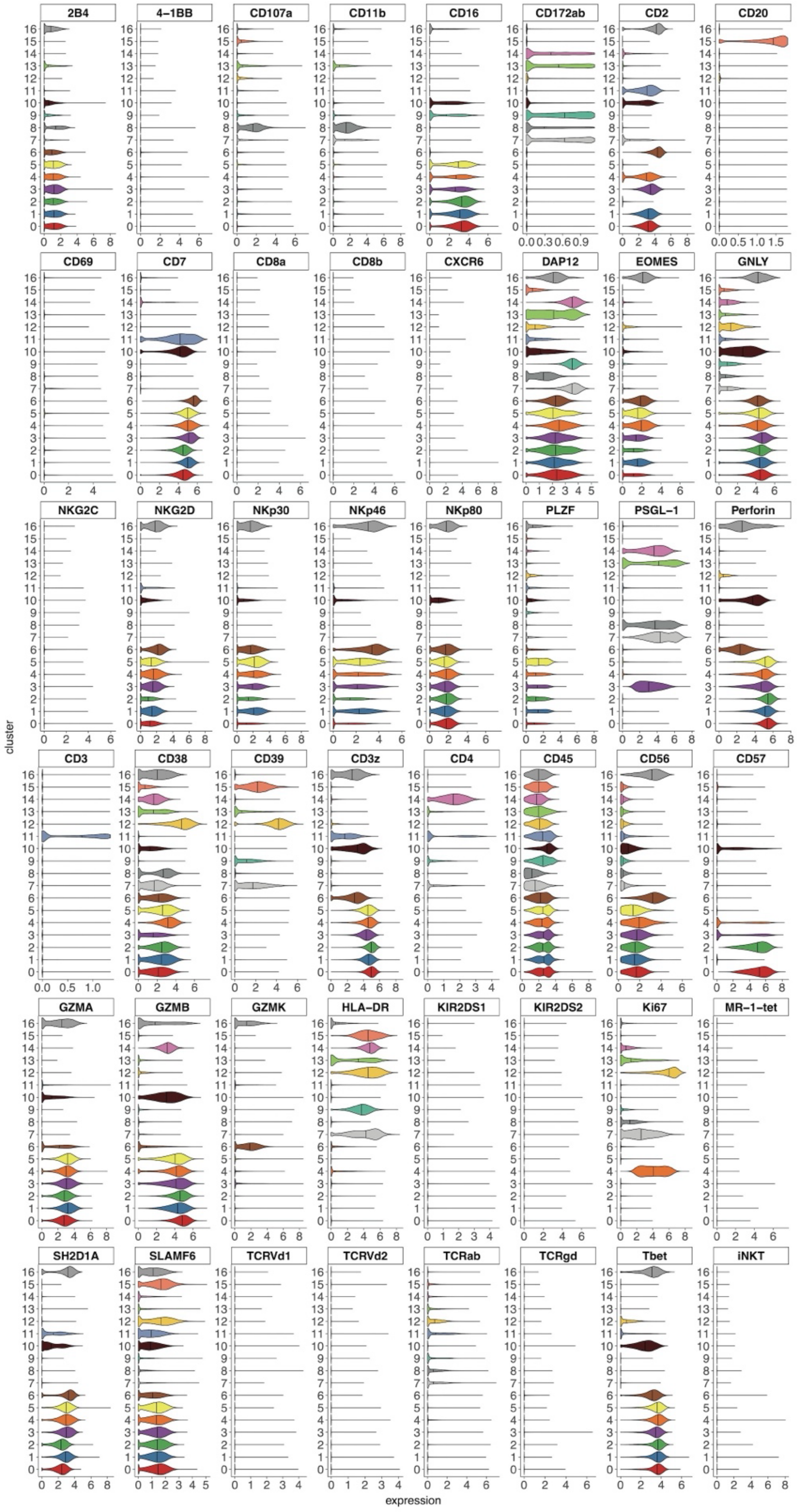

Extended Data Fig. 6: Expression of measured proteins in NK cell panel.

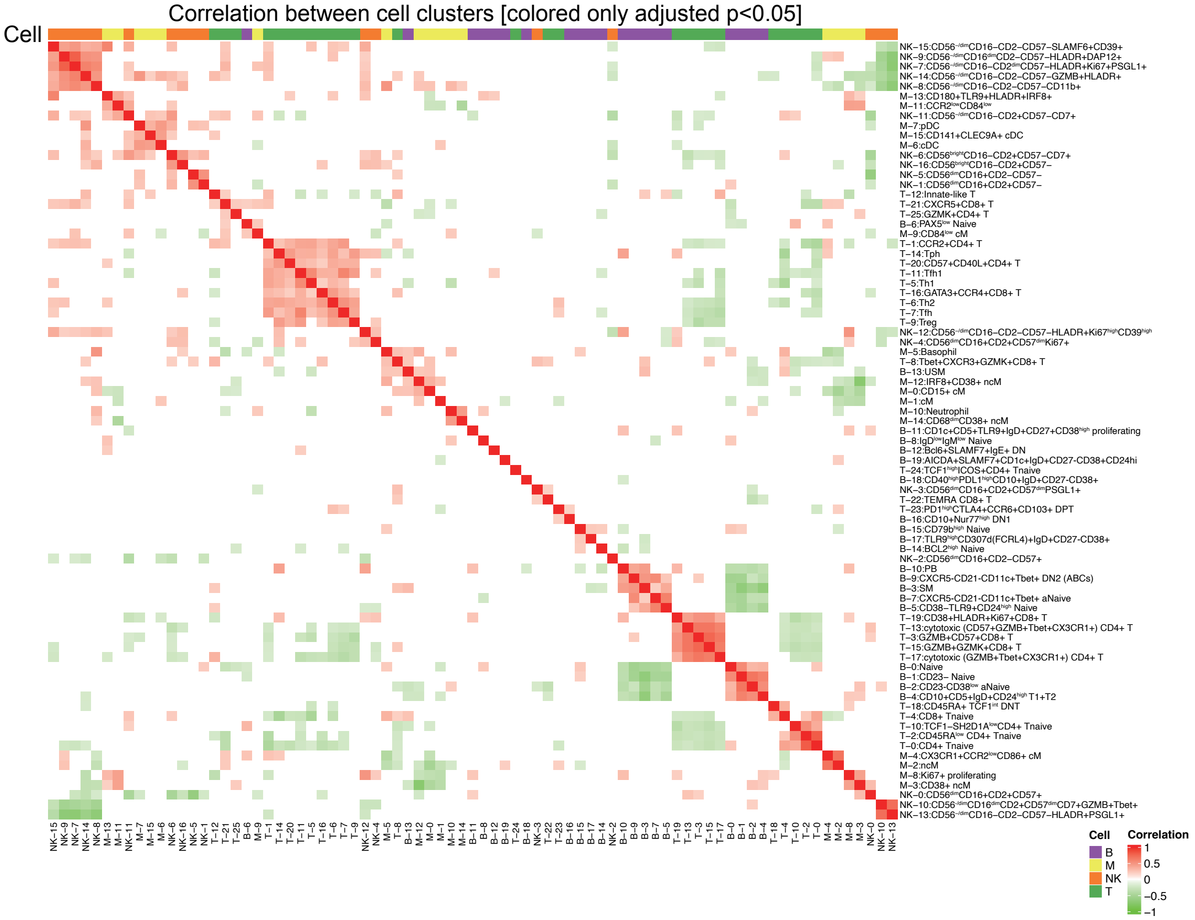

Extended Data Fig. 7: Correlation of abundance in blood between 79 cell types. Correlation plot between cell type abundances. Cells are colored in red (positive) or green (negative) if their false discovery rate is less than 0.05.

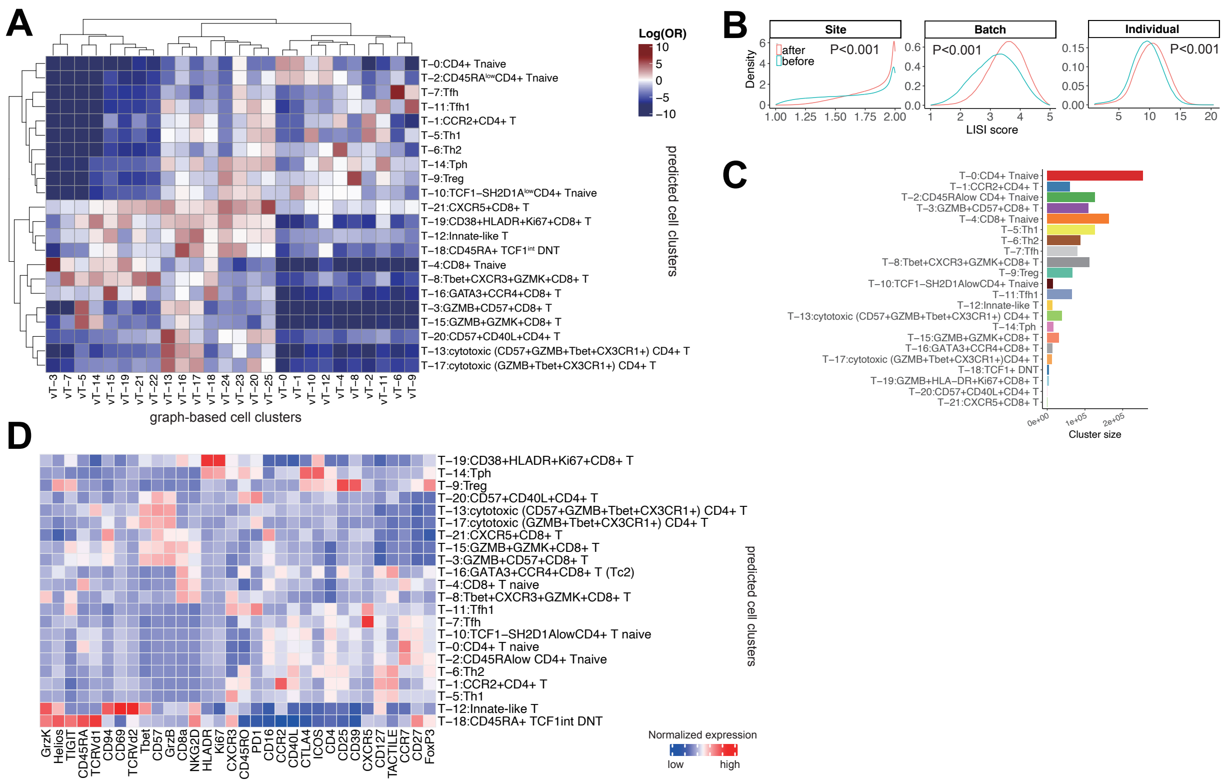

Extended Data Fig. 8: Paired clusters after reference mapping using independent mass cytometry data for T cells. We mapped T cells in the validation dataset onto the corresponding T cell reference from the original T cell panel to determine correspondent cell cluster annotations. **A.** Blue-red color scale in the heatmap indicates the log (OR) for a given pair of states (OR is the ratio of odds of mapping a cell cluster in the validation dataset to a given cluster of the original T cell panel compared to odds of mapping other cells in the validation dataset onto the same cluster of the original T cell panel), with higher values indicating greater correspondence. **B.** LISI scores of T cells from the validation data to measure mixture levels on clinical site, batch, and samples. After batch effect correction, the mixture level of clinical sites (median LISI = 1.85) and technical batches (median LISI = 3.56) are significantly increased compared to before correction (Wilcoxon test $p < 0.01$) suggesting the well mixture of cells in each T cell clusters, **C.** Cell count after assigning predicted cell clusters based on the original T cell panel, **D.** Average expression distributions of variable key proteins in each cluster across samples, scaled within each cell cluster.

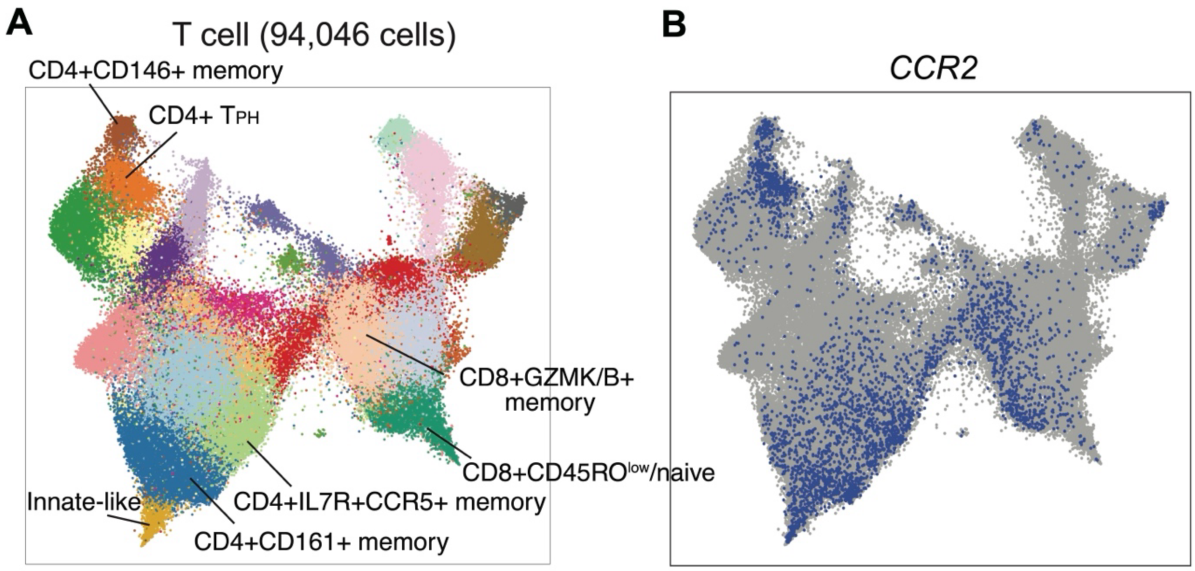

Extended Data Fig. 9: Expression of *CCR2* mRNA in the synovium of RA patients. **A.** T cell clusters identified in the synovium of RA patients in the UMAP space. The annotations of *CCR2*-expressing clusters are labeled. **B.** *CCR2*-expressing cells in the UMAP. Expressing cells are colored in blue.

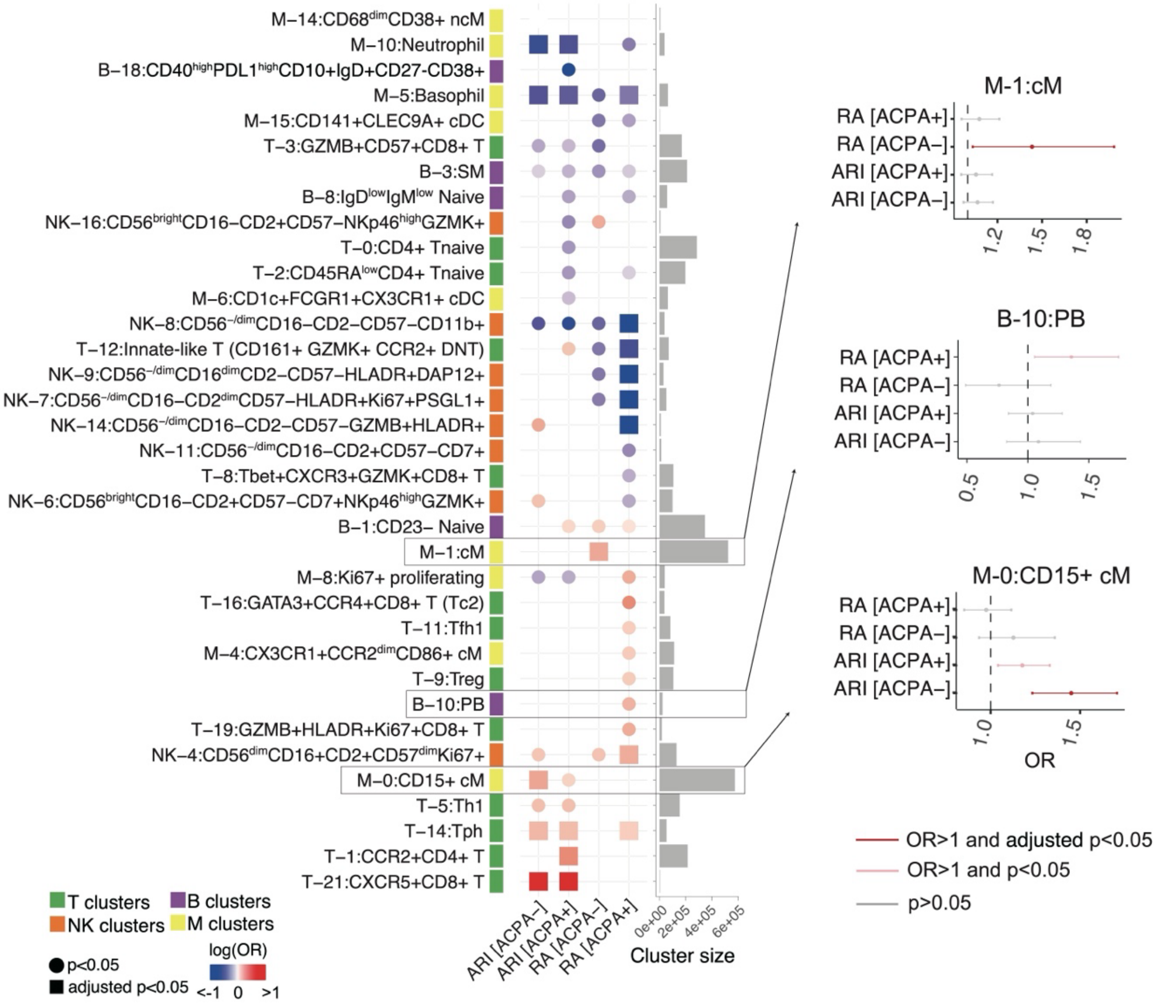

Extended Data Fig. 10: ACPA-status specific analysis reveals unique populations for different disease statuses. Heatmap shows association with each subgroup upon ACPA status in ARI and RA (vs controls) for each cell type. Only clusters with $p < 0.05$ are shown. Circles represent $p < 0.05$ and squares represent adjusted $p < 0.05$. Adjusted p-values were calculated by the Benjamini and Hochberg method. Cell types are colored in red (expanded) or blue (depleted). Error bars on selected cell populations represent 95% confidence intervals. All the results in this analysis are adjusted for age and sex.

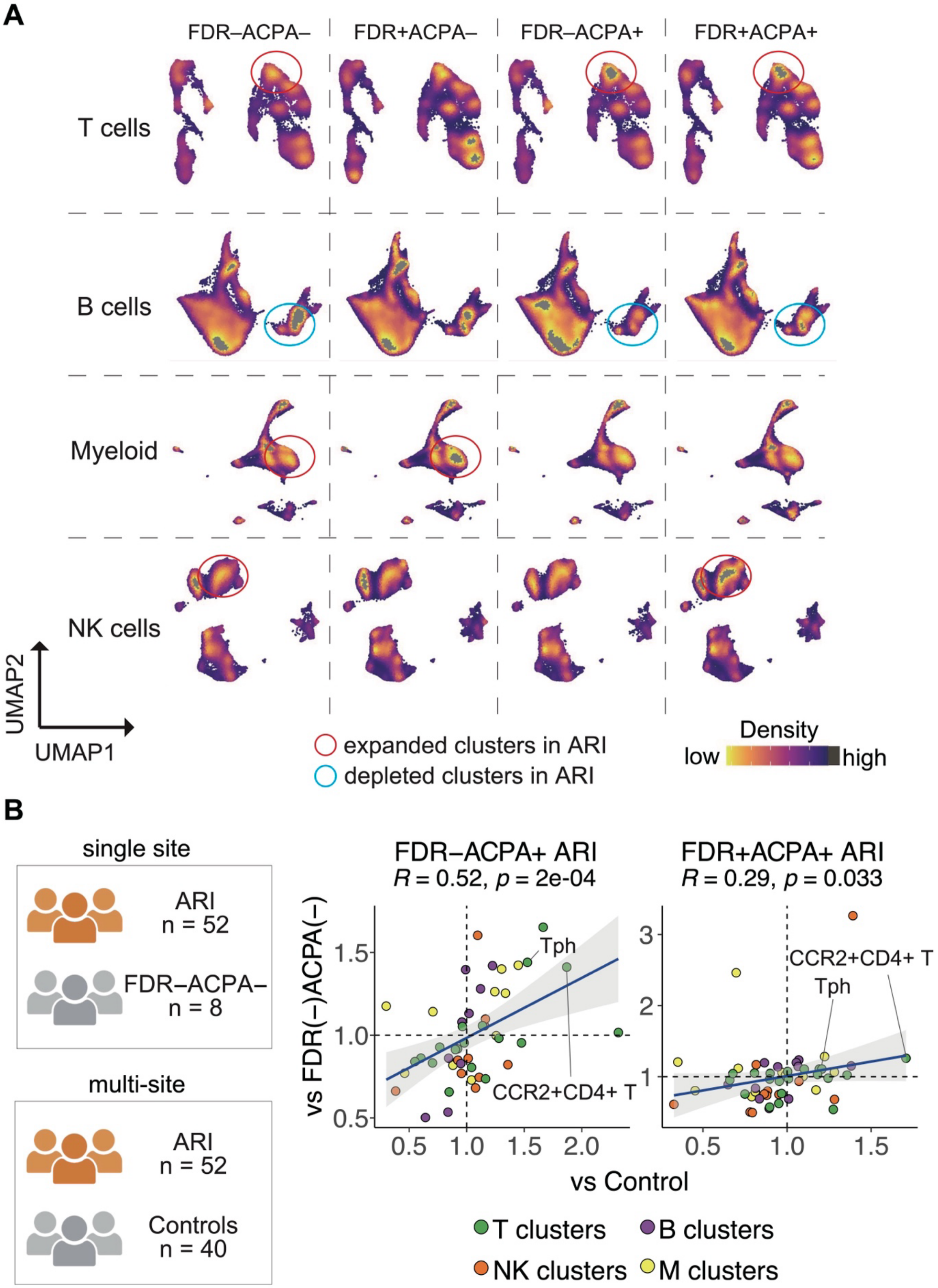
A
FDR–ACPA–
FDR+ACPA–
FDR–ACPA+
FDR+ACPA+
T cells
B cells
Myeloid
NK cells
UMAP2
UMAP1
expanded clusters in ARI
depleted clusters in ARI
Density
low
high
B
single site
ARI
n = 52
FDR–ACPA–
n = 8
multi-site
ARI
n = 52
Controls
n = 40
FDR–ACPA+ ARI
R = 0.52, p = 2e-04
FDR+ACPA+ ARI
R = 0.29, p = 0.033
vs FDR(–)ACPA(–)
vs Control
Tph
CCR2+CD4+ T
T clusters
B clusters
NK clusters
M clusters

Extended Data Fig. 11: Sensitivity analysis for different control groups from multi-clinical sites. A. Density plot by family history and ACPA status according to cell types, **B**. Correlation plot of odds ratios comparing ARI subgroups with FDR−ACPA− controls (y-axis, n=8) from the SERA cohort (y-axis) or healthy controls from other clinical sites (x-axis, n=40). Dots are colored by immune cell types. Of total association tests, 77 cell clusters were included; outliers of the odds ratio (top 99%ile and bottom 1%ile) or size of clusters are lower than 25%ile among all clusters, and results with infinite confidence intervals for the odds ratio were excluded. Statistical results are adjusted for age and sex. Correlation coefficients and p-values were obtained by Spearman's correlation test.

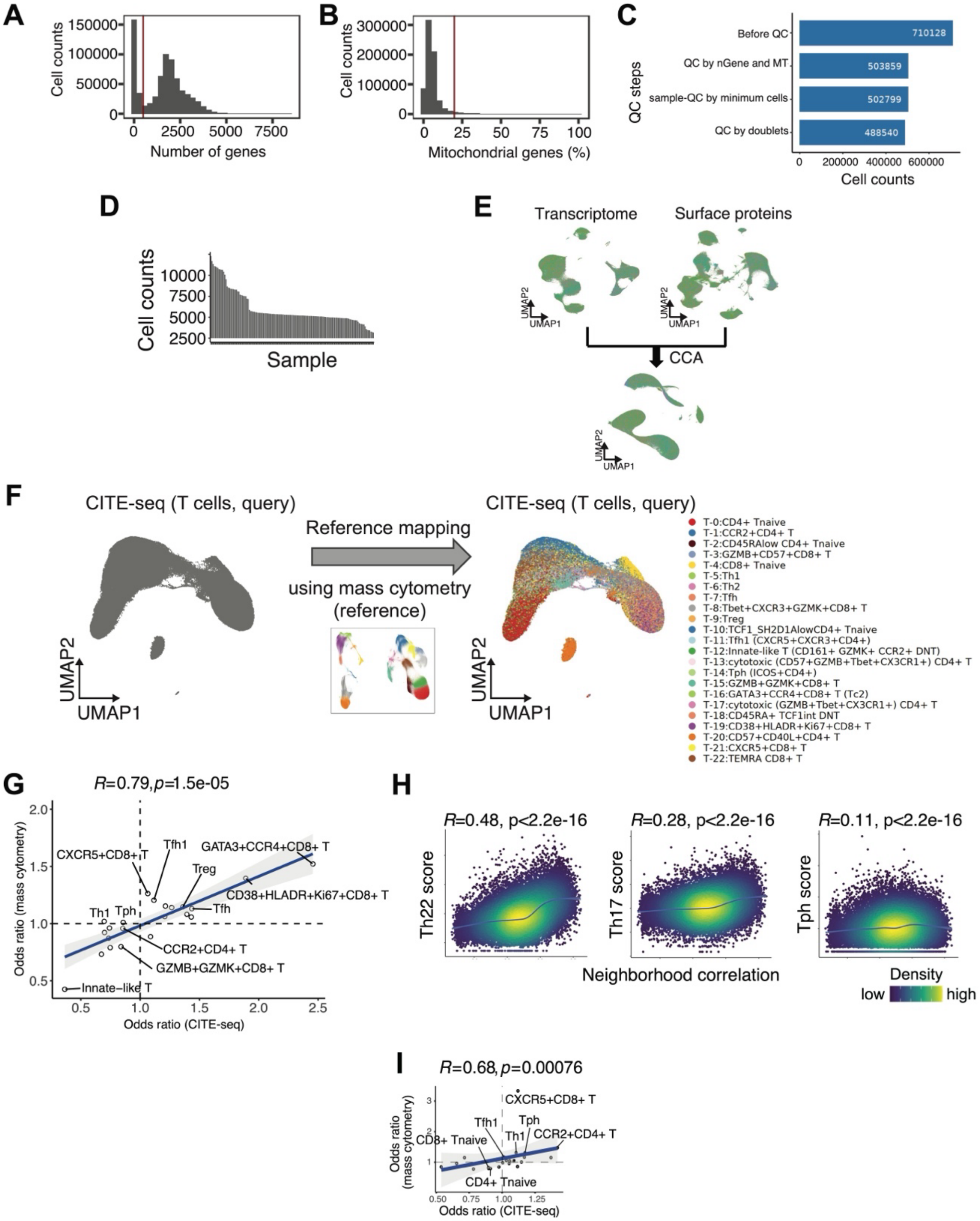

Extended Data Fig. 12. Quality control and processing step for CITE-seq data. A, Distribution of the number of detected genes per cell. The red vertical line represents the threshold used for filtering out low-quality cells based on gene count. **B**, Distribution of the percentage of mitochondrial genes per cell. The red vertical line indicates the threshold used to filter out cells with high mitochondrial content, which is indicative of poor cell quality or stress. **C,** Quality control (QC) steps and their impact on cell count. The bar graph shows the number of cells remaining after each QC step: before QC, after filtering by gene count and mitochondrial

content, after doublet removal, and after sample-level QC to retain samples with a minimum number of cells. **D**, Cell counts per sample after QC steps. Each bar represents the number of cells retained from each sample after quality control. **E**, Integration of transcriptomic and surface protein data using canonical correlation analysis (CCA). UMAP plots show the separate clustering of transcriptomic (left) and surface protein (right) data before integration. The bottom plot shows the integrated dataset with combined clustering of transcriptomic and proteomic data. **F**, Mapping of CITE-seq T cell clusters to mass cytometry reference data. The right UMAP plot shows the reference mapping of CITE-seq T cells using mass cytometry reference data, with clusters annotated according to known T cell subsets. The inset shows the reference mass cytometry data used for mapping. **G**, Scatter plot showing the correlation between odds ratios for patients with RA association for various T cell subsets. Selected cell clusters are labeled. **H**, Scatter plot showing the correlation between ARI association obtained from CNA in **Fig. 7H** and signature scores in **Fig. 7G**. **I**, Scatter plot showing the correlation between odds ratios for ARI association with various T cell clusters. Significantly associated clusters in the mass cytometry analysis are labeled.

We recognize participants in the Accelerating Medicines Partnership® Program: Rheumatoid Arthritis and Systemic Lupus Erythematosus (AMP® RA/SLE) Network, which includes: Jennifer Albrecht[1], William Apruzzese[2], Jennifer L. Barnas[1], Brendan F. Boyce[3], David L. Boyle[4], Debbie Campbell[1], Hayley L. Carr[5], Arnold Ceponis[4], Adam Chicoine[2], Andrew Cordle[6], Michelle Curtis[2,7,8,9], Edward DiCarlo[10], Patrick Dunn[11], Lindsy Forbess[12], Ellen M. Gravallese[2], Peter K. Gregersen[13], Diane Horowitz[13], Lionel B. Ivashkiv[10,14], Gregory Keras[2], Ilya Korsunsky[2,7,8,9], Amit Lakhanpal[14], Katherine P. Liao[2], Zhihan J. Li[2], Yuhong Li[2], Ian Mantel[15], Mark Maybury[16], Mandy J. McGeachy[17], Nida Meednu[1], Alessandra Nerviani[18], Dana E. Orange[10,19], Karim Raza[16], Christopher Ritchlin[1], William H. Robinson[20], Saori Sakaue[2,7,8,9], Melanie H. Smith[10], Dagmar Scheel-Toellner[16], Darren Tabechian[1], Paul J. Utz[20], Michael H. Weisman[12], Zhu Zhu[2].

[1]Division of Allergy, Immunology and Rheumatology, University of Rochester Medical Center, Rochester, NY, United States of America

[2]Department of Medicine, Division of Rheumatology, Inflammation, and Immunity, Brigham and Women's Hospital and Harvard Medical School, Boston, MA, United States of America

[3]Department of Pathology and Laboratory Medicine, University of Rochester Medical Center, Rochester, NY, United States of America

[4]Division of Rheumatology, Allergy and Immunology, University of California, San Diego, La Jolla, CA, United States of America

[5]Rheumatology Research Group, Institute for Inflammation and Ageing, University of Birmingham, Birmingham, United Kingdom

[6]Department of Radiology, University of Pittsburgh Medical Center, Pittsburgh, PA, United States of America

[7]Center for Data Sciences, Brigham and Women's Hospital and Harvard Medical School, Boston, MA, United States of America

[8]Department of Medicine, Division of Genetics, Brigham and Women's Hospital and Harvard

Medical School, Boston, MA, United States of America
[9]Department of Biomedical Informatics, Brigham and Women's Hospital and Harvard Medical School, Boston, MA, United States of America
[10]Department of Pathology and Laboratory Medicine, Hospital for Special Surgery, New York, NY, United States of America
[11]Division of Allergy, Immunology, and Transplantation, National Institute of Allergy and Infectious Diseases, National Institutes of Health, Bethesda, MD, United States of America
[12]Division of Rheumatology, Cedars-Sinai Medical Center, Los Angeles, CA, United States of America
[13]Feinstein Institute for Medical Research, Northwell Health, New York, NY, United States of America
[14]Department of Medicine, Hospital for Special Surgery, New York, NY, United States of America
[15]Weill Cornell Medical College, New York, NY, United States of America
[16]Rheumatology Research Group, Institute for Inflammation and Ageing, University of Birmingham, Birmingham, United Kingdom
[17]Division of Rheumatology and Clinical Immunology, University of Pittsburgh Medical Center, Pittsburgh, PA, United States of America
[18]Centre for Experimental Medicine & Rheumatology, William Harvey Research Institute, Queen Mary University of London and Barts NIHR BRC & NHS Trust, London, United Kingdom
[19]Laboratory of Molecular Neuro-Oncology, The Rockefeller University, New York, NY, United States of America
[20]Division of Immunology and Rheumatology, Institute for Immunity, Transplantation and Infection, Stanford University School of Medicine, Stanford, CA, United States of America
